# SIAH proteins regulate the degradation and intra‐mitochondrial aggregation of PINK1: Implications for mitochondrial pathology in Parkinson's disease

**DOI:** 10.1111/acel.13731

**Published:** 2022-10-28

**Authors:** Fatimah Abd Elghani, Hazem Safory, Haya Hamza, Mor Savyon, Malik Farhoud, Michal Toren‐Hershoviz, Zagorka Vitic, Kirsten Ebanks, Vered Shani, Sleman Bisharat, Lihi Shaulov, Claude Brodski, Zhiyin Song, Rina Bandopadhyay, Simone Engelender

**Affiliations:** ^1^ Department of Biochemistry, Rappaport Faculty of Medicine Technion‐Israel Institute of Technology Haifa Israel; ^2^ Department of Physiology and Cell Biology, Zlotowski Center for Neuroscience, Faculty of Health Sciences Ben‐Gurion University of the Negev Be'er Sheva Israel; ^3^ Reta Lila Weston Institute of Neurological Studies UCL Queen Square Institute of Neurology London UK; ^4^ Hubei Key Laboratory of Cell Homeostasis, College of Life Sciences, Frontier Science Center for Immunology and Metabolism Wuhan University Wuhan China

**Keywords:** intra‐mitochondrial protein aggregation, mitochondrial dysfunction, mitophagy, Parkinson's disease, PINK1, SIAH1, SIAH3, ubiquitin

## Abstract

Parkinson's disease (PD) is characterized by degeneration of neurons, particularly dopaminergic neurons in the substantia nigra. PD brains show accumulation of α‐synuclein in Lewy bodies and accumulation of dysfunctional mitochondria. However, the mechanisms leading to mitochondrial pathology in sporadic PD are poorly understood. PINK1 is a key for mitophagy activation and recycling of unfit mitochondria. The activation of mitophagy depends on the accumulation of uncleaved PINK1 at the outer mitochondrial membrane and activation of a cascade of protein ubiquitination at the surface of the organelle. We have now found that SIAH3, a member of the SIAH proteins but lacking ubiquitin‐ligase activity, is increased in PD brains and cerebrospinal fluid and in neurons treated with α‐synuclein preformed fibrils (α‐SynPFF). We also observed that SIAH3 is aggregated together with PINK1 in the mitochondria of PD brains. SIAH3 directly interacts with PINK1, leading to their intra‐mitochondrial aggregation in cells and neurons and triggering a cascade of toxicity with PINK1 inactivation along with mitochondrial depolarization and neuronal death. We also found that SIAH1 interacts with PINK1 and promotes ubiquitination and proteasomal degradation of PINK1. Similar to the dimerization of SIAH1/SIAH2, SIAH3 interacts with SIAH1, promoting its translocation to mitochondria and preventing its ubiquitin‐ligase activity toward PINK1. Our results support the notion that the increase in SIAH3 and intra‐mitochondrial aggregation of SIAH3‐PINK1 may mediate α‐synuclein pathology by promoting proteotoxicity and preventing the elimination of dysfunctional mitochondria. We consider it possible that PINK1 activity is decreased in sporadic PD, which impedes proper mitochondrial renewal in the disease.

## Graphical abstract

Our study reports that SIAH3 is increased in PD brains. We also found that SIAH3, along with PINK1, is aggregated in the mitochondria of PD brains. The intra‐mitochondrial aggregation of SIAH3 and PINK1 inactivates PINK1, depolarizes the mitochondria, and leads to neuronal death. Pathogenic a‐synuclein pre‐formed fibrils (a‐SynPFF) increase SIAH3 levels and aggregation, suggesting that SIAH3 increase and its mitochondrial aggregation with PINK1 could help mediate a‐synuclein toxicity.

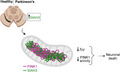

**Abbreviations**
HA: hemagglutinin
PD: Parkinson's disease
PINK1: PTEN‐induced putative kinase 1
SIAH: seven in absentia homolog
siRNA: short interfering RNA

## 1 INTRODUCTION

Parkinson's disease (PD) is a common neurodegenerative disorder. Its most striking neuropathological feature is the presence of inclusions called Lewy bodies and the degeneration of dopaminergic neurons in the substantia nigra pars compacta. Lewy bodies consist mainly of α‐synuclein aggregates and vesicular structures, including mitochondria (Shahmoradian et al., 2019; Spillantini et al., 1997). Lewy bodies occur in sporadic and autosomal dominant forms of the disease, such as mutations in the α‐synuclein gene, LRRK2, VPS35, CHCHD2, among others (Polymeropoulos et al., 1997). On the contrary, dopaminergic neurons degenerate in juvenile PD but do not exhibit robust α‐synuclein accumulation and Lewy bodies (Sasaki et al., 2004). The two major genes mutated in juvenile PD are PINK1 and Parkin, which maintain proper mitochondrial fusion and fission and mitochondrial autophagy (mitophagy) (Arena & Valente, 2017; Kitada et al., 1998; Valente et al., 2004).

PINK1 is a protein kinase localized in mitochondria, and its primary substrates are Parkin and ubiquitin (Kane et al., 2014; Kondapalli et al., 2012; Koyano et al., 2014; Shiba‐Fukushima et al., 2012). Under normal conditions, PINK1 is rapidly imported into mitochondria and processed by mitochondrial proteases that cleave its mitochondrial target sequence at the N‐terminus (Greene et al., 2012; Jin et al., 2010). Upon mitochondrial depolarization, uncleaved full‐length PINK1 accumulates at the outer mitochondrial membrane and allows the recruitment of Parkin. Uncleaved PINK1 at the mitochondrial surface phosphorylates Parkin and ubiquitin, leading to widespread ubiquitination of the mitochondrial surface and activation of mitophagy (Narendra et al., 2010). Mitophagy is critical for degradation of dysfunctional mitochondria and maintenance of mitochondrial function (Narendra et al., 2008, 2010). PINK1 PD mutations inhibit mitophagy, suggesting that dysfunctional mitophagy may play a role in juvenile PD. Mitophagy is also supported by Parkin‐independent PINK1‐dependent pathways, such as synphilin‐1, ARIH1/HHARI, and Vps13D (Shen et al., 2021; Szargel et al., 2016; Villa et al., 2017), suggesting that multiple PINK1 pathways are required to support proper mitophagy.

In addition, several proteins have been shown to modulate the PINK1‐Parkin pathway by increasing Parkin translocation to mitochondria, including AF‐6 and BAG2 (Haskin et al., 2013; Qu et al., 2015). An unbiased siRNA screen identified a SIAH family protein, the mitochondrial protein SIAH3 lacking the ubiquitin‐ligase domain (Robbins et al., 2011), as a negative regulator of the PINK1‐Parkin pathway (Hasson et al., 2013). Although knockdown of SIAH3 leads to an increase in Parkin translocation to mitochondria upon depolarization, the detailed molecular mechanisms by which this occurs are still unclear (Hasson et al., 2013).

We have now found that SIAH3 is elevated in PD brains and in neurons and striatum exposed to preformed α‐synuclein fibrils (α‐synPFF), suggesting that the elevation of SIAH3 may contribute to mediate the pathogenicity of α‐synuclein. We also discovered that elevated SIAH3 in mitochondria co‐aggregates with PINK1 in PD brains and is present in Lewy bodies. In addition, we found that SIAH3 directly interacts with PINK1 and causes aggregation and inactivation of PINK1 in mitochondria, mitochondrial depolarization, and toxicity in cellular and neuronal models. In addition, we found that SIAH1 promotes proteasomal degradation of PINK1 and SIAH3 negatively regulates this process by promoting SIAH1 recruitment to mitochondria and its inactivation. SIAH3 may play a role in the pathogenesis of PD by promoting proteotoxicity and mitochondrial dysfunction. We raise the possibility that inactivation of PINK1 by SIAH3 may contribute to the mitochondrial dysfunction observed in sporadic PD.

## 2 RESULTS

### 2.1 SIAH3 is increased in PD brains and co‐aggregates with PINK1 in the mitochondria in PD

**FIGURE 1:**
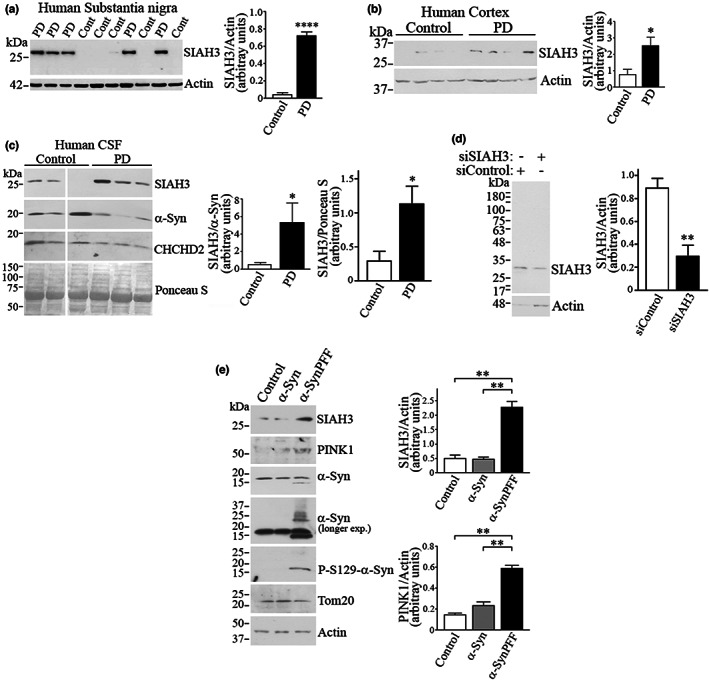
SIAH3 levels are increased in PD brains and CSF. (a,b) Homogenates from substantia nigra (a) or frontal cortex (b) of sporadic PD and matched controls were analyzed with anti‐SIAH3 (upper panel). Graphs depict SIAH3 levels in the nigra (a) and cortex (b) normalized to actin. Figures are the representative of 3 independent Western blot analyses. Values represent the average ± SEM of analyzed samples. *, **** Different from control at *p* < 0.0001 (a), *p* = 0.0235 (b) (Student's *t* test). (c) Aliquotes of CSF of sporadic PD and matched controls were analyzed with anti‐SIAH3, anti‐α‐synuclein and anti‐CHCHD2 antibodies. Ponceau S was used as a loading control. Figure is representative of 3 independent Western blot analyses. Values represent the average ± SEM of analyzed samples. *p = 0.0494 (SIAH3/α‐Syn) and = 0.0425 (SIAH3/Ponceau S) (Student's *t* test). (d) Levels of endogenous SIAH3 in the presence of control siRNA and siRNA to SIAH3 were determined with anti‐SIAH3. Graph depicts SIAH3 levels relative to actin. Values represent the average ± SEM of 4 experiments. **p = 0.0226 (Student's *t* test). (e) α‐SynPFF increases the levels of SIAH3 in neurons. Neurons (DIV4) were incubated with monomeric α‐synuclein and α‐synPFF (2 μg/ml) for 14 days. Graphs represent the levels of SIAH3 and PINK1 relative to actin. Values represent the average ± SEM of 3 independent experiments. ** Different from control at *p* = 0.0013 and 0.002 (SIAH3, control and monomeric, respectively); *p* = 0.0047 and 0.009 (PINK1, control and monomeric, respectively) (Repeated measures one‐way ANOVA with Bonferroni post‐hoc test). Except for the SIAH3 values in the control nigra (a), all human samples have a normal distribution according to the Shapiro–Wilk test (see Supporting Information).

A previous study identified SIAH3 as a mitochondrial protein that inhibits the translocation of Parkin to mitochondria, but the mechanism has remained unclear as SIAH3 lacks ubiquitin‐ligase activity (Hasson et al., 2013). We now sought to investigate the relationship between SIAH3 and PD. We found that SIAH3 levels are increased in the substantia nigra (Figure 1a) and frontal cortex of PD patients (Figure 1b). In addition, SIAH3 and the SIAH3/α‐synuclein ratio are elevated in the CSF of PD patients (Figure 1c). Nevertheless, the analysis of additional CSFs will help to determine the value of SIAH3 as a marker for PD. We confirmed the specificity of the anti‐SIAH3 antibody used by the decrease in signal when cells were transfected with siRNA against SIAH3 (Figure 1d).

We also observed that neurons treated with preformed α‐synuclein fibrils (α‐SynPFF) (characterized by an average size of 10–120 nm by electron microscopy) (Figure S1a), but not with monomeric α‐synuclein, had increased levels of SIAH3 and PINK1 (Figure 1e). Although α‐SynPFF caused some degree of mitochondrial depolarization (Figure S1b), no significant changes in the mitochondrial protein Tom20 were observed (Figure 1e), suggesting that mitophagy is not activated when neurons are exposed to α‐SynPFF, as previously suggested for autophagy (Tanik et al., 2013). Thus, it is possible that pathogenic α‐synuclein promotes a feedforward mechanism of toxicity by increasing SIAH3 levels.

**FIGURE 2:**
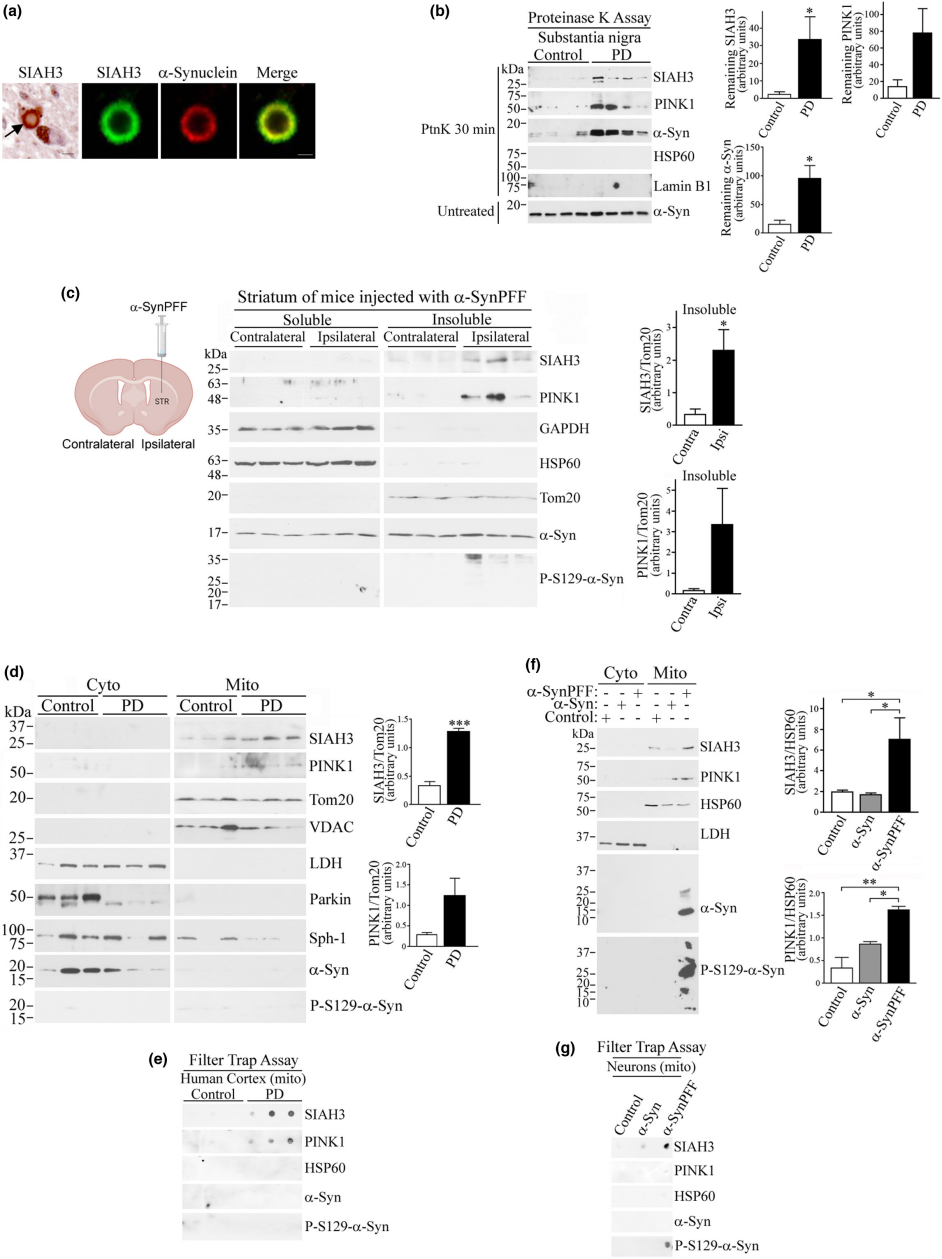
SIAH3, along with PINK1, is aggregated in the mitochondria of PD brains. (a) Human substantia nigra were incubated with anti‐SIAH3 and anti‐α‐synuclein antibodies. The panel at the left represents an adjacent section incubated with anti‐SIAH3 antibody alone and developed by DAB staining. An arrow indicates a SIAH3‐positive Lewy body. Neuromelanine is observed in an adjacent neuron without Lewy bodies. The section was counterstained with Mayer's Haematoxilin. Scale, 5 μm (fluorescent panels) and 10 μm (DAB panel). (b) Homogenates from substantia nigra of PD and controls were incubated with proteinase K for 30 min and probed with indicated antibodies. As control, samples not subjected to proteinase K were probed for α‐synuclein (last panel). Graphs depict remaining SIAH3, PINK1 and α‐synuclein levels after 30 min proteinase K. Figures are representative of 3 independent Western blot analyses. Values represent the average ± SEM of analyzed samples. *p = 0.0485 (SIAH3) and 0.0152 (α‐synuclein), ns = 0.0741 (PINK1) (Student's *t* test). (c) Ipsilateral and contralateral striatum of mice injected unilaterally with α‐SynPFF (30 days) were fractionated into Triton/SDS‐soluble and Triton/SDS‐insoluble fractions. Levels of SIAH3 and PINK1 in the soluble and insoluble fractions were detected with specific antibodies. Differential protein extraction is shown by antibodies used against the indicated proteins. Pathogenicity of α‐SynPFF is shown by the exclusive presence of phosphorylated S129 α‐synuclein in the insoluble fractions of ipsilateral striatum of mice. Figures are the representative of 3 independent Western blot analyses. Values represent the average ± SEM of analyzed samples. *p = 0.0486 (SIAH3), ns *p* = and 0.1390 (PINK1) (Student's *t* test). (d) Cytosolic and mitochondrial fractions from human frontal cortex of control and PD brains. The Levels of lSIAH3 and PINK1 in mitochondria were detected with specific antibodies. The purity of the fractionation was determined by the levels of Tom20, VDAC, and LDH. Graphs represent the relative levels of SIAH3 and PINK1 in mitochondrial fractions corrected by the levels of Tom20. Figures are the representative of 3 independent Western blot analyses. Values represent the average ± SEM of analyzed samples. ***p = 0.0004 (SIAH3) and 0.0854 (PINK1) (Student's *t* test). (e) Purified mitochondria from human frontal cortex of control and PD brains were analyzed by cellulose acetate filter (filter trap assay) using specific antibodies to indicated proteins. (f) Cytosolic and mitochondrial fractions from neurons treated with monomeric α‐synuclein and α‐SynPFF. The levels of SIAH3 and PINK1 in mitochondria were detected with specific antibodies. The purity of fractionation was determined by the levels of HSP60 and LDH. Graphs represent the relative levels of SIAH3 and PINK1 in mitochondrial fractions corrected by the levels of HSP60. Values represent the average ± SEM of 3 independent experiments. *p = 0.0378 (SIAH3, control × α‐SynPFF) and 0.0217 (SIAH3, monomeric × α‐SynPFF) and *, **p = 0.0019 (PINK1, control × α‐SynPFF) and 0.0254 (PINK1 monomeric × α‐SynPFF) (Repeated measures one‐way ANOVA with Bonferroni post‐hoc test). (g) Purified mitochondria from neurons exposed to monomeric α‐synuclein and α‐SynPFF were analyzed by cellulose acetate filter (filter trap assay) using specific antibodies to indicated proteins. All human and mouse samples have a normal distribution according to the Shapiro–Wilk test (see Supporting Information).

Because PD is considered a proteinopathy (Engelender & Isacson, 2017; Spillantini et al., 1997), we investigated whether SIAH3 can aggregate in PD. First, we found that SIAH3 is present in 8%–22% of midbrain Lewy bodies of five PD patients (Figure 2a). Similar to α‐synuclein, we found that SIAH3 was resistant to proteinase K treatment in the brains of PD (Figure 2b), supporting the idea that increased SIAH3 leads to its aggregation in PD. We also found that PINK1 also showed a clear trend toward resistance to proteinase K treatment (Figure 2b).

We next injected α‐SynPFF in the striatum of mice to investigate if pathogenic α‐synuclein can cause SIAH3 aggregation in vivo. We found that SIAH3 was significantly increased in 1% Triton X‐100/0.1% SDS insoluble fractions of ipsilateral but not contralateral striatum after one month of α‐SynPFF injection (Figure 2c). PINK1 showed a clear trend toward an increase in insoluble fractions of ipsilateral striatum injected with α‐SynPFF (Figure 2c). The pathological process was confirmed by the presence of phosphorylated S129 α‐synuclein only in insoluble fractions of ipsilateral striatum (Figure 2c).

Consistent with the predicted mitochondrial localization, SIAH3 is increased in mitochondrial fractions from PD patients compared with control subjects (Figure 2d). Although detection of endogenous PINK1 by conventional PAGE‐SDS is difficult (Gandhi et al., 2006; Muqit et al., 2006), we found that PINK1 also tended to be increased in mitochondria from PD using a specific PINK1 antibody that we developed previously (Szargel et al., 2016) (Figure 2d). In addition, SIAH3 and PINK1 aggregated in mitochondrial fractions from PD brains but not from control patients (Figure 2e), as determined by cellulose acetate filter‐trap assays (Scherzinger et al., 1997). The specificity of SIAH3 and PINK1 aggregation in mitochondria from PD patients is supported by the fact that the mitochondrial chaperone HSP60 is not retained in the filter‐trap assay (Figure 2e). We also failed to detect α‐synuclein and phosphorylated S129‐α‐synuclein in the cellulose acetate filter of mitochondria from PD (Figure 2e). These results suggest specific aggregation of SIAH3 and PINK1 in mitochondria during disease progression.

Next, we found that α‐SynPFF increases the levels and aggregation of SIAH3 in purified mitochondria of neurons (Figure 2f,g). An increase in PINK1 levels in mitochondria was also observed (Figure 2f), but PINK1 was not retained in cellulose acetate filters (Figure 2g). The pathogenic process of α‐SynPFF was confirmed by aggregation of phosphorylated S129 α‐synuclein (Figure 2f,g). These results suggest that aggregated α‐synuclein in mitochondria may initially cause SIAH3 aggregation, which leads to PINK1 aggregation as the disease progresses, as confirmed by the aggregation of PINK1 in mitochondria from PD patients (Figure 2e).

### 2.2 Characterization of intra‐mitochondrial aggregation of SIAH3 PINK1 in cells and neurons

**FIGURE 3:**
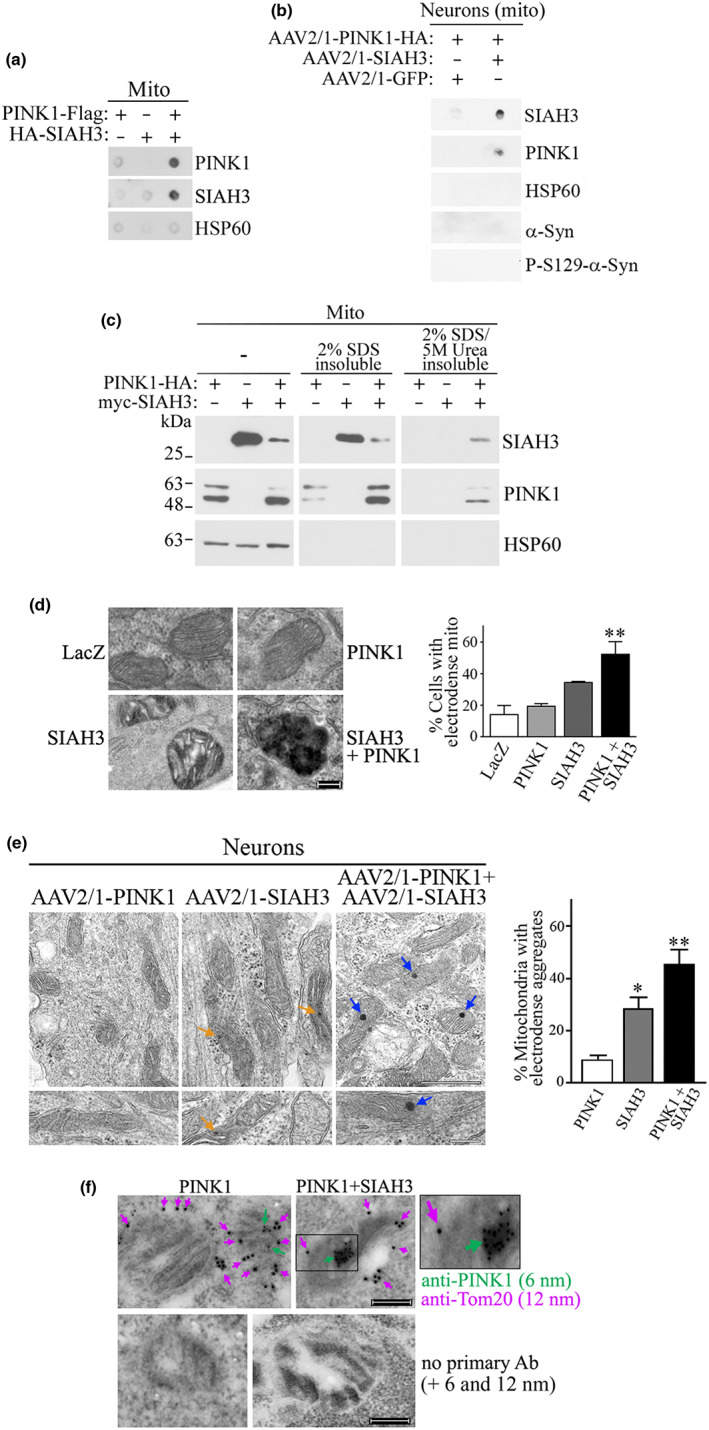
SIAH3 promotes PINK1 aggregation inside the mitochondria of cells and neurons. (a) Mitochondrial fractions were purified from transfected HEK293 cells (a) and neurons (b). The degree of protein aggregation in the mitochondria was determined by cellulose acetate filter (filter trap assay). In A, aggregation of SIAH3 and PINK1 was determined using anti‐HA and anti‐Flag, respectively. In B, SIAH3 and PINK1 aggregation was determined with anti‐SIAH3 and anti‐HA, respectively. (c) Mitochondrial fractions from transfected HEK293 cells were treated with 2% SDS followed by addition of 5M urea. Insoluble pellets of each condition were subjected to Western blot. Levels of SIAH3 and PINK1 were determined with anti‐myc and anti‐HA, respectively. Mitochondrial loading was determined with anti‐HSP60. (d,e) Transmission electron microscopy of transfected HEK293 cells (d) and AAV2/1‐transduced neurons (e) showing aggregate formation inside mitochondria when in the presence of SIAH3 and PINK1. Blue arrows show condensed aggregates by PINK1 and SIAH3 in neurons (right panels). Orange arrows show smaller and less dense aggregates by SIAH3 in neurons (middle panels). Scale, 200 nm (D), 500 and 200 nm (e). Values are the average ± SEM of 3 independent experiments. ** Different from control at *p* = 0.0042 (d) and * and ** *p* = 0.0311 and 0.0015, respectively (e) (Repeated measures one‐way ANOVA with Bonferroni post‐hoc test). (f) Immunoelectron microscopy depicts aggregation of PINK1 in the mitochondria by SIAH3. Transfected HEK293 cells were assayed with anti‐PINK1 (green arrows) and anti‐Tom20 (purple arrows). Inset depicts PINK1 aggregate within the mitochondria. Lower panels depict the lack of 6 and 12 nm gold staining when in the absence of primary antibodies. Scale, 200 nm.

**FIGURE 4:**
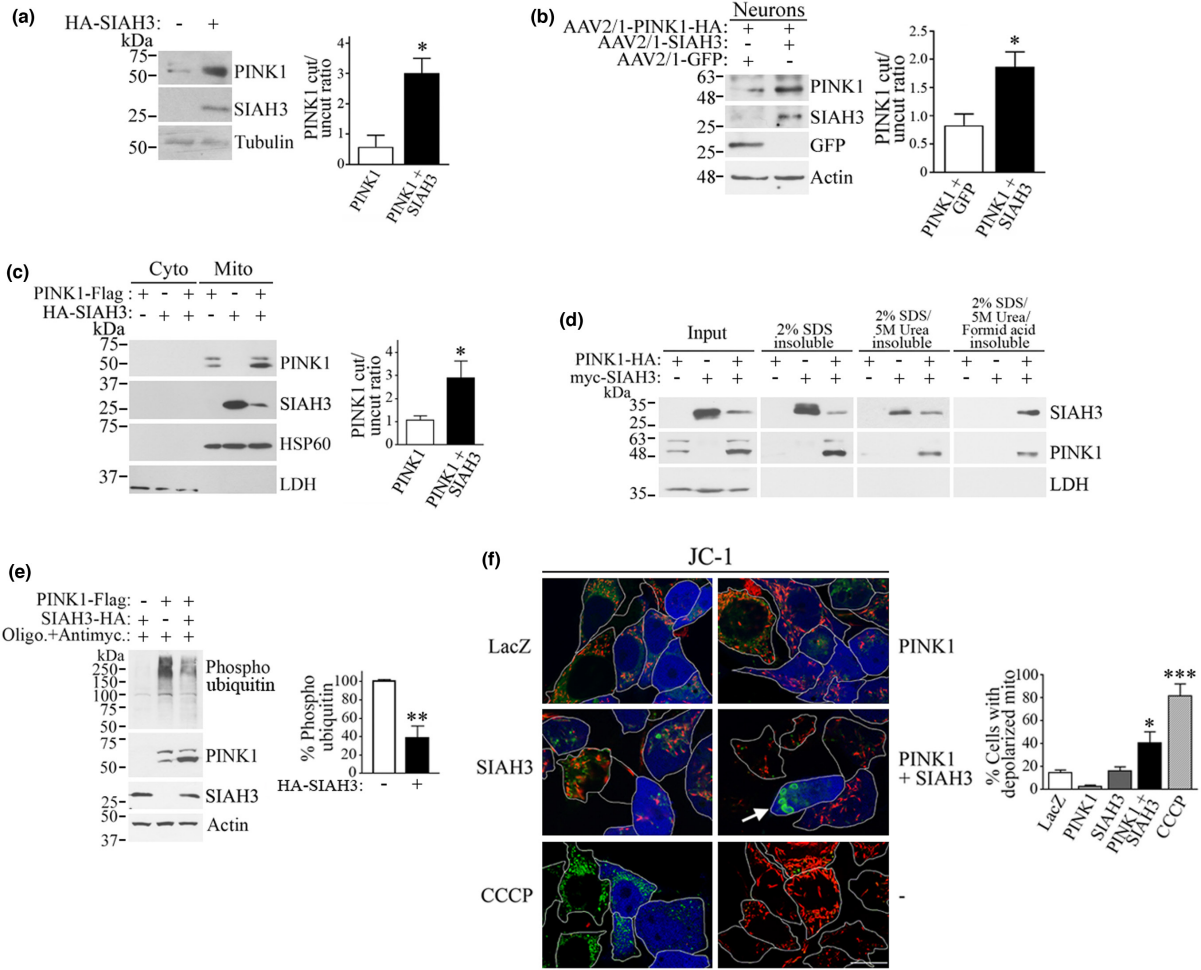
Aggregation promoted by SIAH3 leads to PINK1 inactivation in the mitochondria. (a) HEK293 cells were transfected with HA‐SIAH3, and cleavage of endogenous PINK1 was determined with anti‐PINK1 (upper panel). Graph represents the ratio of cleaved over uncleaved endogenous PINK1. *p = 0.039 (Student's *t* test). (b) Neurons transduced with AAV2/1 particles driven by synapsin promoter were analyzed for PINK1 cleavage using anti‐HA. *p = 0.0367 (Student's *t* test). (c) Cell lysates of transfected HEK293 cells were fractionated into cytosolic and mitochondrial fractions. PINK1 and SIAH3 were monitored using anti‐Flag and anti‐HA, respectively. The purity of cytosolic and mitochondrial fractions was examined using anti‐LDH and anti‐HSP60, respectively. Graph represents the ratio of cleaved over uncleaved PINK1. *p = 0.031 (Student's *t* test). (d) Transfected HEK293 cells were treated with 2% SDS followed by 5M urea and 70% formic acid. Insoluble pellets of each condition were subjected to Western blot. Levels of SIAH3 and PINK1 were determined with anti‐myc and anti‐HA, respectively. LDH was used as loading control. (e) Transfected HEK293 cells were treated for 12 h with 4 μM antimycin, 10 μM oligomycin, and 20 μM QVD (Szargel et al., 2016). Phosphorylation of ubiquitin was analyzed using anti‐phospho‐ubiquitin. Levels of PINK1 and SIAH3 were determined using anti‐Flag and anti‐HA, respectively. Graph represents the percentage of phosphorylated ubiquitin in cells. **p = 0.0064 (Student's *t* test). (f) Transfected HEK293 cells were incubated with JC‐1 and analyzed by live microscopy. Red JC‐1 corresponds to polarized, whereas green JC‐1 represents depolarized mitochondria (arrow). Transfected cells (BFP‐positive cells) are outlined, and a cell with green only JC‐1 (depolarized) in the presence of SIAH3 and PINK1 is indicated by an arrow. Scale, 10 μm. Graph represents the percentage of transfected cells with depolarized mitochondria (with green only JC‐1). *, *** Different from control at *p* = 0.0323 and = 0.0001, respectively (Repeated measures one‐way ANOVA with Bonferroni post‐hoc test). Values in all graphs represent the average ± SEM of 3 independent experiments.

We next characterized the ability of SIAH3 and PINK1 expression to form aggregates inside the mitochondria. We found that SIAH3 and PINK1 are retained in the cellulose acetate filter when expressed together in the mitochondria of cells and neurons (Figure 3a,b). Neither SIAH3 nor PINK1 alone was retained in the filter‐trap assay (Figure 3a,b). Also, HSP60 was not aggregated when examined by the filter‐trap assay, even when in the presence of SIAH3 and PINK1 (Figure 3a,b). Moreover, α‐synuclein and phosphorylated S129 α‐synuclein in mitochondria of neurons were not retained in the filter‐trap assay upon aggregation of SIAH3 and PINK1 (Figure 3b). The purity of the mitochondrial preparation is shown in Figure 4c. These results support the data obtained with PD tissues (Figure 2) where SIAH3 and PINK1, but not all mitochondrial proteins, co‐aggregate in mitochondria.

We next extracted purified mitochondria with high SDS concentration and urea. We found that SIAH3 and PINK1 aggregates were resistant to a combination of 2% SDS and 5M urea extraction (Figure 3c). Neither SIAH3 nor PINK1 alone was resistant to the addition of urea (Figure 3c), confirming that SIAH3 and PINK1 together form resistant aggregates in mitochondria.

To further characterize the co‐aggregation of SIAH3 and PINK1 in mitochondria, we conducted electron microscopic analysis in a cellular context. We observed that cells expressing SIAH3 and PINK1 exhibited a striking accumulation of condensed electron‐dense material within mitochondria (Figure 3d). SIAH3 alone exhibited only a low level of electron‐dense material within the organelle (Figure 3d), suggesting that endogenous PINK1 may co‐aggregate with increased SIAH3. Under more physiological conditions, when neurons were transduced with AAV2/1‐SIAH3 and AAV2/1‐PINK1 under the synapsin promoter, we also observed the formation of electron‐dense material within the mitochondria of neurons (Figure 3e). SIAH3 alone also resulted in some aggregate formation. However, these aggregates were much smaller and less condensed than those formed in combination with PINK1 (Figure 3e).

We next performed immunoelectron microscopy experiments and found that PINK1, when expressed alone, was distributed near the mitochondrial membrane with no evidence of aggregation (Figure 3f, upper left panel, arrowhead). In contrast, in the presence of SIAH3, PINK1 was localized in aggregates within mitochondria (Figure 3f, upper right panel, arrow). The specificity of the signal was confirmed by analysis of cells in the absence of primary antibody, in which no gold‐labeled signal was detected (Figure 3f, lower panels).

We next examined the consequences of intra‐mitochondrial aggregation of SIAH3 and PINK1. We observed an increase in PINK1 levels and cleavage in cells and neurons (Figure 4a,b). In particular, we observed that SIAH3 promotes the accumulation of cleaved PINK1 in mitochondria (Figure 4c). Interestingly, we found that SIAH3 levels decreased in the presence of PINK1 (Figure 4c), which is likely due to the strong aggregation of SIAH3 in the presence of PINK1, as its signal is enriched in lysates treated with formic acid (Figure 4d).

Consistent with the accumulation of cleaved PINK1, we found that SIAH3 reduced PINK1 kinase activity relative to its bona fide substrate ubiquitin (Kane et al., 2014; Kazlauskaite et al., 2014; Koyano et al., 2014) (Figure 4e). Moreover, the expression of SIAH3 and PINK1, but not PINK1 or SIAH3 alone, promoted the accumulation of depolarized and clustered mitochondria (arrow) (Figure 4f).

**FIGURE 5:**
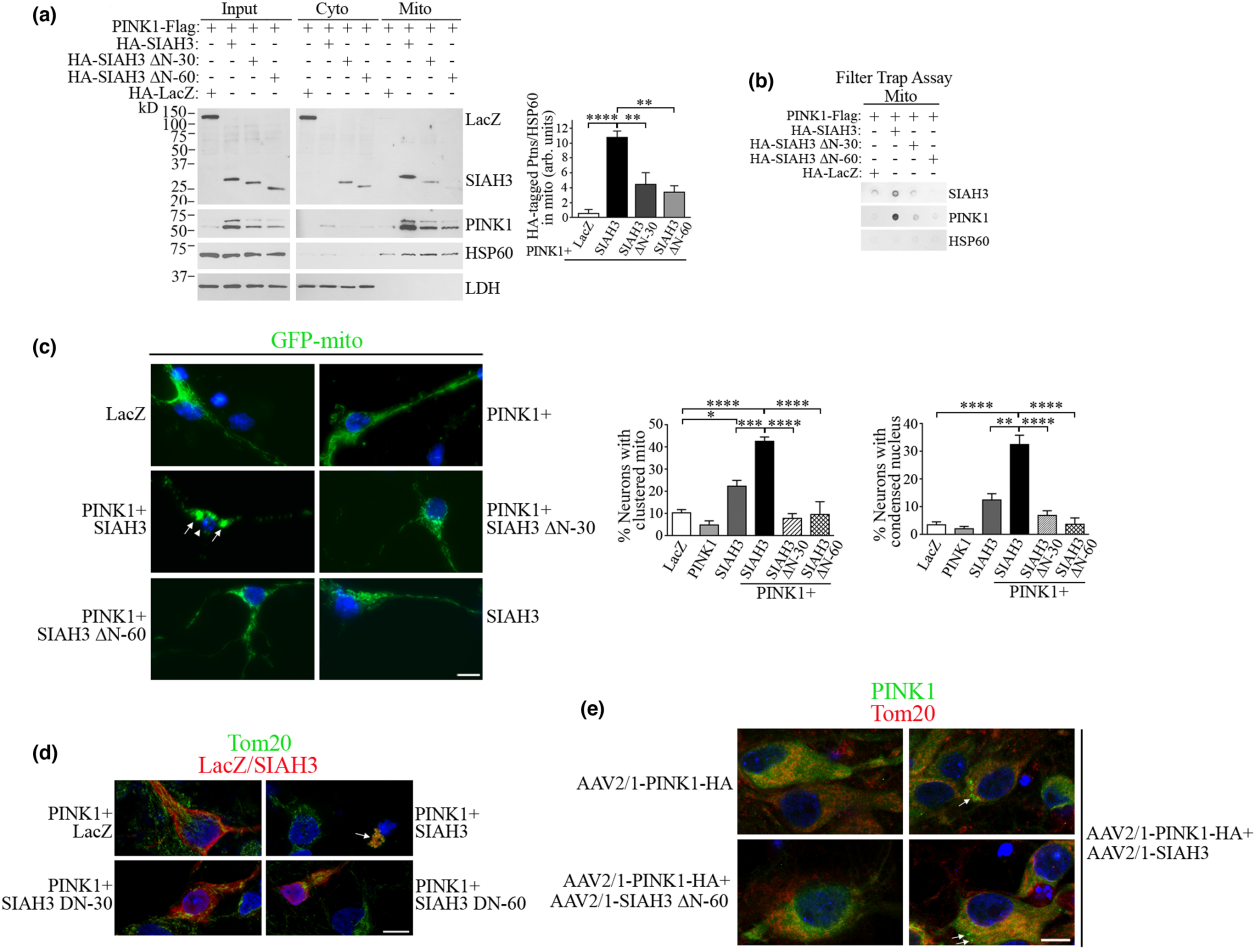
SIAH3 and PINK1 co‐aggregation in mitochondria leads to neuronal toxicity. (a) Mitochondria of transfected HEK293 cells were fractionated, and levels of SIAH3 constructs were determined with anti‐HA. Purity of fractions was determined by anti‐HSP60 and anti‐LDH. Graph represents the levels of HA‐tagged proteins in mitochondria relative to HSP60. ****, ** Different from control at *p* < 0.0001; *p* = 0.0055 and 0.0017 (ΔN‐30 and ΔN‐60, respectively) (Repeated measures one‐way ANOVA with Bonferroni post‐hoc test). (b) Mitochondrial fractions from transfected HEK293 cells were analyzed by filter trap assay. The degree of SIAH3 constructs and PINK1 aggregation were determined with anti‐HA and anti‐Flag, respectively. (c) Neurons were transfected with PINK1‐Flag in the presence of HA‐SIAH3 or HA‐LacZ. Neurons were analyzed for mitochondrial (GFP‐mito) clustering and nuclear condensation and fragmentation. Arrows indicate mitochondrial clustering when in the presence of SIAH3 and PINK1. Scale, 10 μm. Graphs depict the percent of GFP‐mito‐positive neurons containing clustered mitochondria and condensed and fragmented nuclei. For mitochondrial clustering, different from control at *, ****p = 0.0318 and <0.0001, respectively. For nuclear condensation, **, **** different from control at *p* = 0.0015 and <0.0001, respectively (Repeated measures one‐way ANOVA with Bonferroni post‐hoc test). (d) Neurons were transfected with PINK1‐Flag and HA‐SIAH3 constructs and analyzed with anti‐HA (red) and anti‐Tom20 (green). Arrow indicates the presence of full‐length SIAH3 in clustered mitochondria when in the presence of PINK1. Scale, 10 μm. (e) Neurons were transduced with AAV2/1‐PINK1‐HA in the presence of AAV2/1‐SIAH3 or AAV2/1‐SIAH3 ΔN‐60 and analyzed with anti‐HA (green) and anti‐Tom20 (red). Arrows indicate the presence of PINK1‐positive clusters when in the presence of SIAH3 but not SIAH3 ΔN‐60. Scale, 10 μm. Values in all graphs represent the average ± SEM of 3 independent experiments.

To further investigate the specificity of SIAH3 and PINK1 aggregation in mitochondria, we generated truncated SIAH3 constructs lacking the N‐terminal region encoding a putative mitochondrial targeting signal. Accordingly, deletion of the first 30 (ΔN‐30) and especially the 60 amino acids (ΔN‐60) significantly reduced translocation of SIAH3 to mitochondria (Figure 5a). Using these constructs, we found that PINK1 was retained with full‐length SIAH3, but not with SIAH3 ΔN‐30 or ΔN‐60, in the filter‐trap assay (Figure 5b), indicating that SIAH3 aggregates with PINK1 only when it is in the mitochondria.

We next examined the aggregation of SIAH3 and PINK1 in the mitochondria of neurons. We observed that PINK1, together with full‐length SIAH3 but not SIAH3 ΔN‐30 and ΔN‐60, leads to mitochondrial clustering in neurons, as visualized by the mitochondrial marker GFP‐mito (Figure 5c and graph). Full‐length SIAH3 aggregated in mitochondrial clustering in neurons, whereas SIAH3 truncations without mitochondrial targeting did not (Figure 5d). Concurrent with mitochondrial clustering, SIAH3 and PINK1 promote neuronal death, as observed by DNA condensation and fragmentation when nuclei are stained with Hoechst 33342 (Figure 5c and graph). Under more physiological conditions in which neurons were transduced with AAV under the synapsin promoter, we also observed aggregation of PINK1 in the presence of SIAH3 but not SIAH3 ΔN‐60 (Figure 5e). These results suggest that intra‐mitochondrial aggregation of SIAH3 with PINK1 in neurons leads to mitochondrial clustering and neuronal toxicity.

### 2.3 Interaction of SIAH3 and SIAH1 with PINK1

**FIGURE 6:**
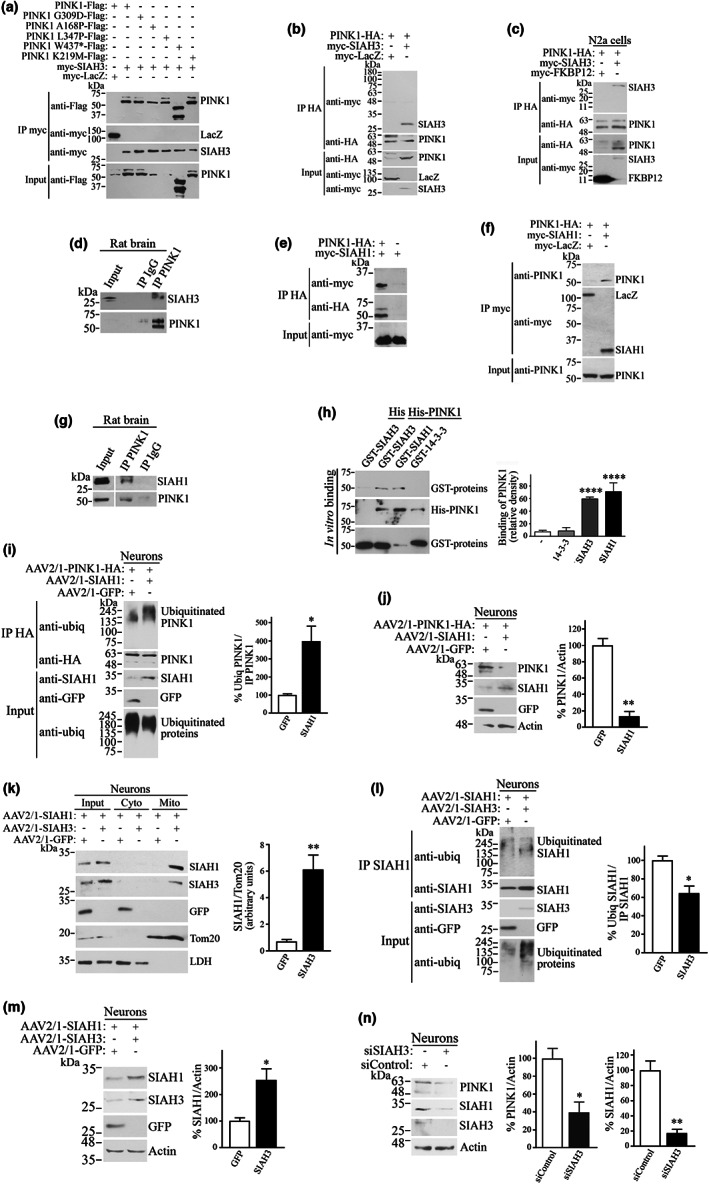
SIAH1 promotes ubiquitination and degradation of PINK1 and SIAH3 hinders this process. (a) Co‐immunoprecipitation of PINK1 with SIAH3. Transfected HEK293 cell lysates were subjected to immunoprecipitation with anti‐myc. Co‐immunoprecipitation of PINK1 was detected with anti‐Flag. Immunoprecipitation was detected with anti‐myc. (b) Co‐immunoprecipitation of HA‐tagged PINK1 with SIAH3. Transfected HEK293 cell lysates were subjected to immunoprecipitation with anti‐HA. Co‐immunoprecipitation of SIAH3, but not LacZ, was detected with anti‐myc. Immunoprecipitation of PINK1 was detected with anti‐HA. (c) Co‐immunoprecipitation of PINK1 with SIAH3 in N2a cells. Transfected N2a cell lysates were processed for immunoprecipitation with anti‐HA. Co‐immunoprecipitation of SIAH3, but not the control FKBP12, was detected with anti‐myc. Immunoprecipitation of PINK1 was detected with anti‐HA. (d) Endogenous PINK1 was immunoprecipitated from brain lysate using anti‐PINK1 (lower panel), and co‐immunoprecipitation of SIAH3 with PINK1, but not with IgG, was determined with anti‐SIAH3 (upper panel). (e) Co‐immunoprecipitation of SIAH1 with PINK1. Transfected HEK293 cell lysates were subjected to immunoprecipitation with anti‐HA. Co‐immunoprecipitation of SIAH1 was detected with anti‐myc. Immunoprecipitation of PINK1 was detected with anti‐HA. (f) Reverse assay shows the co‐immunoprecipitation of PINK1 (anti‐PINK1) with SIAH1, but not with the control LacZ (anti‐myc). (g) PINK1 and SIAH1 interact in vivo. PINK1 was immunoprecipitated from rat brains using anti‐PINK1 antibody (lower panel), and co‐immunoprecipitation was determined with anti‐SIAH1 antibody (upper panel). (h) Direct binding of recombinant purified PINK1 and SIAH3 as well as SIAH1. Purified GST‐proteins (SIAH3, SIAH1 and 14‐3‐3) were incubated with His‐PINK1, and binding was determined using anti‐GST antibody (first panel). Levels of added His‐PINK1 (second panel) and GST‐proteins (third panel) were determined by anti‐His and anti‐GST antibodies, respectively. Graph represents the binding of SIAH3, SIAH1, and 14‐3‐3 proteins to His‐PINK1 compared to that of His alone. **** Different from control at *p* < 0.0001 (Repeated measures one‐way ANOVA with Bonferroni post‐hoc test). (i) SIAH1 promotes the ubiquitination of PINK1 in neurons. AAV2/1‐transduced neurons were treated with 10 μM lactacystin for 16 h. PINK1‐HA was immunoprecipitated with anti‐HA, and ubiquitination of the immunoprecipitate was determined with anti‐ubiquitin. Graph shows the percent of ubiquitinated PINK1‐HA relative to the levels of immunoprecipitated PINK1‐HA. *p = 0.0226 (Student's *t* test). (j) SIAH1 decreases PINK1 steady‐state levels in neurons. AAV2/1‐transduced neurons were analyzed for the levels of PINK1 using anti‐HA. Graph depicts the percent of PINK1‐HA relative to actin. **p = 0.0011 (Student's *t* test). (k) SIAH3 promotes the translocation of SIAH1 to the mitochondria of neurons. Mitochondrial fractions from transduced neurons were analyzed, and the presence of SIAH1 was determined with anti‐SIAH1 (first panel). Levels of SIAH3 in mitochondria was detected with anti‐SIAH3 (second panel). Fractionation purity was determined by Tom20 and LDH. Graph represents the levels of SIAH1 in mitochondrial fractions corrected to the levels of Tom20, in the absence and presence of SIAH3. **p = 0.0077 (Student's *t* test). (l) Decrease of SIAH1 auto‐ubiquitination by SIAH3 in neurons. Levels of SIAH1 ubiquitination were determined with anti‐ubiquitin (first panel). Immunoprecipitated SIAH1 was detected with anti‐SIAH1 (second panel). Graph represents the percent of ubiquitinated SIAH1 relative to the levels of immunoprecipitated SIAH1. *p = 0.0169 (Student's *t* test). (m) Accumulation of SIAH1 steady‐state by SIAH3 in neurons. Levels of SIAH1 in AAV2/1‐transduced neurons were determined with anti‐SIAH1 (upper panel). Graph depicts the percent of SIAH1 relative to actin, in the presence of GFP and SIAH3. *p = 0.0253 (Student's *t* test). (n) Neurons were transfected with siRNA control and siRNA to SIAH3. Levels of PINK1 and SIAH1 were determined with anti‐PINK1 and anti‐SIAH1, respectively. Graphs represent the percent of PINK1 and SIAH1 relative to actin, in the presence of siControl and siSIAH3. *, **p = 0.0204 and 0.033, respectively (Student's *t* test). Values in all graphs represent the average ± SEM of 3 independent experiments.

Although the authors did not find an interaction between SIAH3 and PINK1 in the first screening study (Hasson et al., 2013), our results suggest otherwise. The possibility of an interaction between the two proteins in mitochondria leading to their aggregation is supported by the much lower aggregation of SIAH3 and PINK1 alone (Figure 3). We first found that PINK1 wild‐type and PINK1 disease mutants interact with SIAH3 in cells as they co‐immunoprecipitate from cell lysates (Figure 6a). Co‐immunoprecipitation also occurs when PINK1 is HA‐tagged and N2a cells are used (Figure 6b,c). SIAH3 and PINK1 interact in vivo, as SIAH3 co‐immunoprecipitates with PINK1, but not with control IgG, from brain tissues (Figure 6d).

Because SIAH3 is a homolog of the ubiquitin‐ligases SIAH1/2 (Robbins et al., 2011), we sought to investigate whether SIAH1 can also interact with PINK1. Consistent with this, we found that SIAH1 specifically co‐immunoprecipitates with PINK1 (Figure 6e) and vice‐versa (Figure 6f). Using rat brain tissues, we observed that SIAH1 co‐immunoprecipitates with PINK1 but not with IgG (Figure 6g), indicating that PINK1 and SIAH1 interact in vivo.

We next investigated if the interaction of SIAH3 and SIAH1 with PINK1 is direct by carrying out in vitro binding experiments with purified proteins only. We found that both GST‐SIAH3 associates with His‐PINK1 but not with His alone (Figure 6h). In addition, GST‐SIAH1, but not GST‐14‐3‐3, interacts with His‐PINK1 (Figure 6h). Altogether, our results suggest that PINK1 interacts directly with SIAH3 and SIAH1 and that aggregation of SIAH3 with PINK1 in mitochondria depends on their direct interaction in the organelle.

### 2.4 Ubiquitination by SIAH1 targets PINK1 for proteasomal degradation

Next, we found that SIAH1 increases the ubiquitination of PINK1 in neurons and cells (Figures 6i and S2a). This effect was observed only in the presence of the proteasome inhibitor lactacystin (Figures 6i and S2a,b) but not in the presence of the lysosome and autophagy inhibitors ammonium chloride and 3‐MA, respectively (Figure S2b), indicating that polyubiquitination by SIAH1 targets PINK1 for proteasomal degradation. The knockdown of SIAH1 by siRNA decreases PINK1 ubiquitination (Figure S2c), supporting the notion that SIAH1 functions as a ubiquitin‐ligase for PINK1.

We next investigated whether ubiquitination by SIAH1 leads to proteasomal degradation of PINK1. We found that SIAH1 decreases the steady‐state levels of PINK1 in neurons (Figure 6j). Similarly, SIAH1 and SIAH2, but not the control GFP, strongly decrease the steady‐state levels of PINK1 in cells (Figure S3a), and this was prevented by lactacystin (Figure S3b). In contrast, the ubiquitin‐ligase Parkin, which is known to interact with PINK1 (Narendra et al., 2008), does not decrease the steady‐state levels of PINK1 (Figure S3a). In addition, catalytically inactive SIAH1 (C55A, H59A, C72S; SIAH1 DN) (Liani et al., 2004) and knockdown of SIAH1 by siRNA significantly increase the steady‐state levels of PINK1 (Figure S3c,d), suggesting that endogenous SIAH1 regulates PINK1 levels.

SIAH1 also decreased the steady‐state levels of PINK1 disease mutants (Figure S3e) and interacted with PINK1 disease mutant G309 in a similar manner observed in the wild‐type (Figure S3f). Finally, the reduction in PINK1 levels by SIAH1 is not due to a reduction in transcription because cycloheximide‐chase experiments show that SIAH1 significantly increases the rate of PINK1 degradation (Figure S3g). Therefore, our results suggest that endogenous SIAH1 promotes ubiquitination and proteasomal degradation of PINK1.

### 2.5 SIAH3 promotes the translocation of SIAH1 to mitochondria, preventing its ubiquitin‐ligase activity

SIAH1 homodimerizes and heterodimerizes with SIAH2 (Polekhina et al., 2002). Therefore, we investigated the possibility that SIAH3 could also dimerize with SIAH1 and affect PINK1 degradation. In agreement, we found that both SIAH1 (and SIAH2) interact with SIAH3 as they specifically co‐immunoprecipitate (Figure S4a). As a consequence of this interaction, SIAH3 promotes the translocation of SIAH1 to mitochondria in neurons and cells (Figures 6k and S4b), resulting in the decreased ubiquitin‐ligase activity of SIAH1, as shown by the decrease in SIAH1 auto‐ubiquitination (Figures 6l and S4c) and the increase in SIAH1 own steady‐state levels in neurons and cells (Figures 6m and S4d).

The decrease in SIAH1 ubiquitin‐ligase activity by SIAH3 also results in decreased PINK1 ubiquitination (relative to immunoprecipitated PINK1 levels) (Figure S4e). This decrease in PINK1 ubiquitination by SIAH3 leads to the accumulation of total PINK1 levels (Figure S4f). Thus, inhibition of SIAH1 in mitochondria by SIAH3 differs from that observed in the PINK1‐synphilin‐1 pathway, where translocation of SIAH1 by direct interaction with synphilin‐1 promotes general ubiquitination of outer membrane mitochondrial proteins (Szargel et al., 2016).

Further evidence for the role of SIAH3 in inhibiting SIAH1 activity is the fact that the expression of SIAH3 increases endogenous levels of SIAH1 and PINK1 (Figure S4g), whereas knockdown of SIAH3 decreases both levels in neurons and cells (Figures 6n and S4h). Remarkably, SIAH3 increased the levels of cleaved endogenous PINK1 (Figure S4g), as was observed with exogenous PINK1 (Figure 4c). Finally, the accumulation of cleaved PINK1 and aggregation by SIAH3 seems to occur in the mitochondria as the knockdown of SIAH3 in neurons does not change the translocation of PINK1 to the mitochondria (Figure S4i).

## 3 DISCUSSION

PINK1 is important for maintaining mitochondrial homeostasis and mitophagy (Arena & Valente, 2017). Although familial mutations in PINK1 prevent its ability to promote mitophagy, the status of PINK1 activity in sporadic PD is unclear. We have now found that sporadic PD brains and CSF have elevated levels of SIAH3. The administration of α‐SynPFF to neurons and striatum of mice increased the levels of SIAH3 and PINK1. SIAH3 and, to a lesser extent, PINK1 aggregated in the striatum of mice injected with α‐SynPFF. Consistent with these findings, SIAH3 and PINK1 co‐aggregate in the mitochondria of neurons and PD brains, a finding consistent with their direct interaction that we now describe. Moreover, intra‐mitochondrial aggregation of SIAH3 and PINK1 in cells and neurons leads to the accumulation of inactive, cleaved PINK1 within the organelle, resulting in the buildup of depolarized mitochondria and neuronal toxicity. We hypothesize that intra‐mitochondrial aggregates represent a novel aspect of the pathogenesis of PD downstream of and mediated by α‐synuclein. Our results suggest that PINK1 activity may be decreased in the disease, contributing to the accumulation of defective mitochondria in PD.

PINK1 is a short‐lived protein that is degraded by the proteasome. This explains the low levels of PINK1 in the human brain and the paucity of sensitive antibodies for its detection (Gandhi et al., 2006; Muqit et al., 2006; Szargel et al., 2016). Despite these limitations, PINK1 levels, especially the processed form, were shown to be increased in PD brains, particularly in the substantia nigra (Muqit et al., 2006). We also found a trend toward increased PINK1 levels in PD mitochondrial fractions, although its activity is probably reduced because of the aggregation we observed with SIAH3.

Ubiquitination and proteasomal degradation of PINK1 was shown to occur via the N‐end rule (Yamano & Youle, 2013) and ER‐associated degradation (Guardia‐Laguarta et al., 2019). We found that endogenous SIAH1 leads to ubiquitination and proteasomal degradation of PINK1. Thus, SIAH1 not only promotes the ubiquitination of other PD proteins, including α‐synuclein and LRRK2 (Rott et al., 2008; Shani et al., 2019) but now also represents a ubiquitin ligase that promotes degradation of PINK1, most likely in the course of its translocation to mitochondria.

We now show that SIAH3 heterodimerizes with SIAH1, promoting its translocation to the mitochondria and inhibiting its ubiquitin‐ligase activity. This inactivation is consistent with our report showing that SIAH1 activity is inhibited once it translocates to the nucleus after binding to LRRK2 (Shani et al., 2019). On the contrary, translocation of SIAH1 to mitochondria promoted by synphilin‐1 is required for the ubiquitination of mitochondrial outer membrane proteins (Szargel et al., 2016). Although the extent of synphilin‐1 translocation to mitochondria in PD requires further investigation, we believe it is possible that increased levels of SIAH3 in PD promote a net decrease in SIAH1 ubiquitin‐ligase, contributing to proteotoxicity and reduction of mitophagy in the disease.

Although the knockdown of SIAH3 was shown to increase the translocation of Parkin to mitochondria by depolarizing agents, the authors failed to identify an interaction between PINK1 and SIAH3 (Hasson et al., 2013). In contrast, we found that SIAH3 directly interacts with and inactivates PINK1. In this framework, SIAH3 and PINK1 aggregates inside the mitochondria may prevent PINK1 from reaching the outer membrane of the organelle, leading to the accumulation of dysfunctional mitochondria. Thus, silencing SIAH3 may increase the net availability of PINK1 to carry out its kinase activity at the mitochondrial surface when needed. Additional studies are required to determine the possible deleterious role of SIAH3 and PINK1 co‐aggregation in vital mitochondrial functions, such as energy production and mitochondrial protein import.

Our data suggest that the aggregation of SIAH3 and PINK1 in mitochondria is specific and not due to their mere overexpression. This is supported by SIAH3 constructs lacking a mitochondrial target sequence that do not translocate to mitochondria and do not promote aggregation of PINK1 in the organelle. In addition, under more physiological conditions, we observed aggregation of SIAH3 and PINK1 in mitochondria in neurons transduced with AAV particles under the synapsin promoter. Of note, the intra‐mitochondrial aggregates of SIAH3 and PINK1 are abundant in neurons but less voluminous than the aggregates observed in HEK293 cells, likely due to the lower ability of the synapsin promoter to induce protein expression compared with that of the CMV promoter. Moreover, under our experimental conditions, the expression of PINK1 alone in cells and neurons did not promote its substantial aggregation in mitochondria or spontaneous activation of mitophagy.

The accumulation of dysfunctional mitochondria is recognized as a factor in the pathogenesis of PD (Schapira et al., 1989), and mitochondria were shown to be an intrinsic part of Lewy bodies (Shahmoradian et al., 2019). The presence of SIAH3 in Lewy bodies may indicate that mitochondria with aggregated SIAH3 are not excluded from Lewy bodies. Still, no clear inhibitory mechanism for PINK1 has been proposed in sporadic PD. The prevailing notion is that the lysosomal and autophagy machinery may be impaired and unable to promote mitophagy (Dehay et al., 2010). Our results suggest that increased SIAH3 may contribute to PINK1 inactivation and the accumulation of stale mitochondria in PD. Therefore, therapeutic attempts to enhance autophagy/lysosomal pathways may not be sufficient to restore mitochondrial function, and strategies to lower SIAH3 levels may help improve mitophagy in PD.

Several studies have examined intra‐mitochondrial aggregates that occur under various circumstances, including hypoxia, aging, and the knockdown of mitochondrial proteases (Kaufman et al., 2017). Although the consequences of protein aggregation within the organelle are still unclear, our findings align with recent studies suggesting that intra‐mitochondrial aggregates can be detrimental to cells. Supporting this notion, the accumulation of a phenylalanine dipeptide (FF) with a mitochondria‐targeting moiety (Mito‐FF) within mitochondria leads to organelle dysfunction through membrane disruption and apoptosis (Jeena et al., 2017). We raise the possibility that aggregation of cleaved PINK1 in mitochondria during disease may prevent it from being translocated back to the cytosol (Takatori et al., 2008), which exacerbates the accumulation of PINK1 in the organelle and is consistent with the PINK1 aggregation we observe in mitochondria of PD brains.

It is long known that pathogenic α‐synuclein promotes mitochondrial dysfunction (Tanaka et al., 2001). Although this process is likely multifaceted, some studies have suggested that α‐synuclein may directly affect mitochondria (Di Maio et al., 2016). Also, α‐SynPFF was reported to translocate to the mitochondria and decrease oxidative phosphorylation (Wang et al., 2019). We observed that both SIAH3 and PINK1 levels were increased with α‐SynPFF treatment and that SIAH3 and, to a lesser extent, PINK1 aggregated. The explanation for these findings is not clear, but it may be due to the interaction of aggregated phosphorylated α‐synuclein with mitochondria, leading to a cascade of deleterious events, including mitochondrial depolarization, which we now show, and reduction in mitochondrial oxidative phosphorylation (Wang et al., 2019). Consistent with this possibility, we observe that phosphorylated α‐synuclein is aggregated in mitochondrial fractions of α‐SynPFF‐treated neurons and in the striatum of mice treated with α‐SynPFF.

Moreover, our findings suggest that the interaction of pathogenic α‐synuclein with mitochondria may represent an early event in the disease, as it is detected in the organelle of α‐SynPFF models but not in the mitochondria of PD. On the contrary, mitochondrial aggregation of SIAH3 is triggered by α‐SynPFF, and full pathology of PINK1 is observed in later stages of α‐synuclein pathology in PD brains, suggesting that co‐aggregation of SIAH3 and PINK1 in the mitochondria may represent a downstream process to pathogenic α‐synuclein. It is unclear how pathogenic α‐synuclein is removed from mitochondria during the disease. Further experiments with longer incubation times of α‐SynPFF in mouse brains and detailed analysis of mitochondria will help elucidate the dynamics of accumulation and aggregation of SIAH3 and PINK1 compared with α‐synuclein in the organelle in PD.

Our study shows that SIAH3 is increased and aggregated in the mitochondria of PD along with PINK1. We also observed that aggregated PINK1 in mitochondria is less active and cannot properly clear depolarized mitochondria. We hypothesize that α‐synuclein may mediate mitochondrial damage by leading to the co‐aggregation of SIAH3 and PINK1 in the mitochondria during the course of the disease, making the organelle less available for mitophagy and contributing to its accumulation in sporadic PD. Moreover, the inactivation of SIAH1 upon translocation to the mitochondria promoted by SIAH3 may contribute to the proteotoxicity in the disease. SIAH3 now represents a novel target for improving mitochondrial function and proteostasis in PD.

## 4 MATERIALS AND METHODS

### 4.1 Mitochondrial preparation

Mitochondria were isolated using a discontinuous Percoll gradient, as described (Haskin et al., 2013). Briefly, brain tissues, neurons, and cells were disrupted using a glass homogenizer in buffer containing 250 mM sucrose, 20 mM Hepes, 3 mM EDTA, 20 mM sodium fluoride, 2 mM sodium orthovanadate, 10 mM inorganic pyrophosphate, 20 mM β‐glycerol phosphate, 30 μM MG132, and a protease inhibitor cocktail (Complete, Roche). Nucleus, membranes, and debris were discarded after low‐speed centrifugation. Supernatant was then spun at 7000× *g* for 10 min, and the crude mitochondrial pellet was resuspended in 15% Percoll in cold homogenizing buffer, layered onto a discontinuous Percoll gradient of 23% over 40%, and then centrifuged for 20 min at 73,000× *g* at 4°C. The interface band between the 23% and 40% Percoll layers was collected and washed twice in cold homogenizing buffer. The mitochondrial pellet was suspended in homogenizing buffer.

A detailed description of the additional procedures utilized in the study is described in SI Materials and Methods.

## AUTHOR CONTRIBUTIONS

Fatimah Abd Elghani, Hazem Safory, Vered Shani, Sleman Bisharat, and Simone Engelender conceived or designed the work. Fatimah Abd Elghani, Hazem Safory, Haya Hamza, Mor Savyon, Malik Farhoud, Michal Toren‐Hershoviz, Zagorka Vitic, Kirsten Ebanks, Vered Shani, Sleman Bisharat, and Lihi Shaulov contributed data or analysis tools. Fatimah Abd Elghani, Hazem Safory, Kirsten Ebanks, Claude Brodski, Zhiyin Song, Rina Bandopadhyay, and Simone Engelender performed the analysis. Fatimah Abd Elghani, Hazem Safory, Claude Brodski, Zhiyin Song, Rina Bandopadhyay, and Simone Engelender wrote and edited the manuscript.

## CONFLICT OF INTEREST

None declared.

## Supporting information


Figure S1

Figure S2

Appendix S1

Appendix S1

Appendix S1

Appendix S1

## Data Availability

The datasets generated in the current study are available from the corresponding author on reasonable request.
