# Supplementary figures and images for: SIAH proteins regulate the degradation and intra‐mitochondrial aggregation of PINK1: Implications for mitochondrial pathology in Parkinson's disease
Source: Aging Cell. 2022 Oct 28;21(12):e13731. doi: 10.1111/acel.13731 (PMC9741505; doi:10.1111/acel.13731)

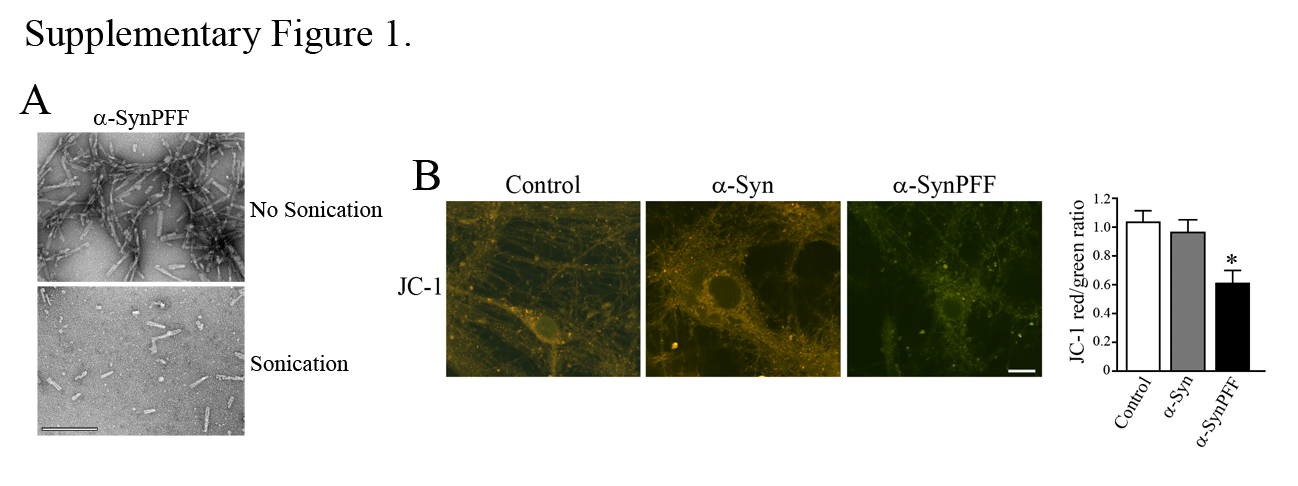

Supplement: Supplementary file 1 — Figure S1 [file ACEL-21-e13731-s004.tif]

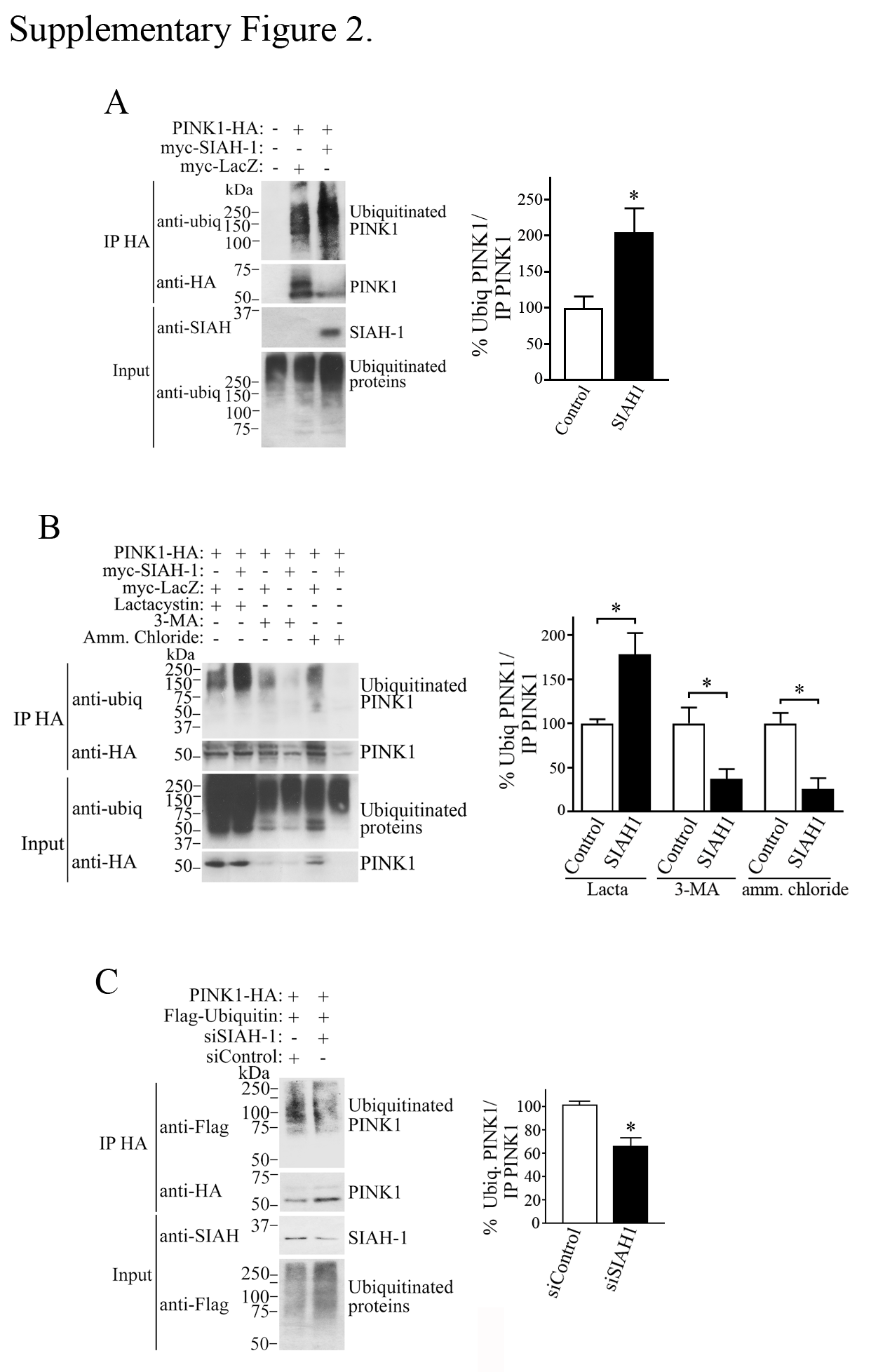

Supplement: Supplementary file 2 — Figure S2 [file ACEL-21-e13731-s006.tif]

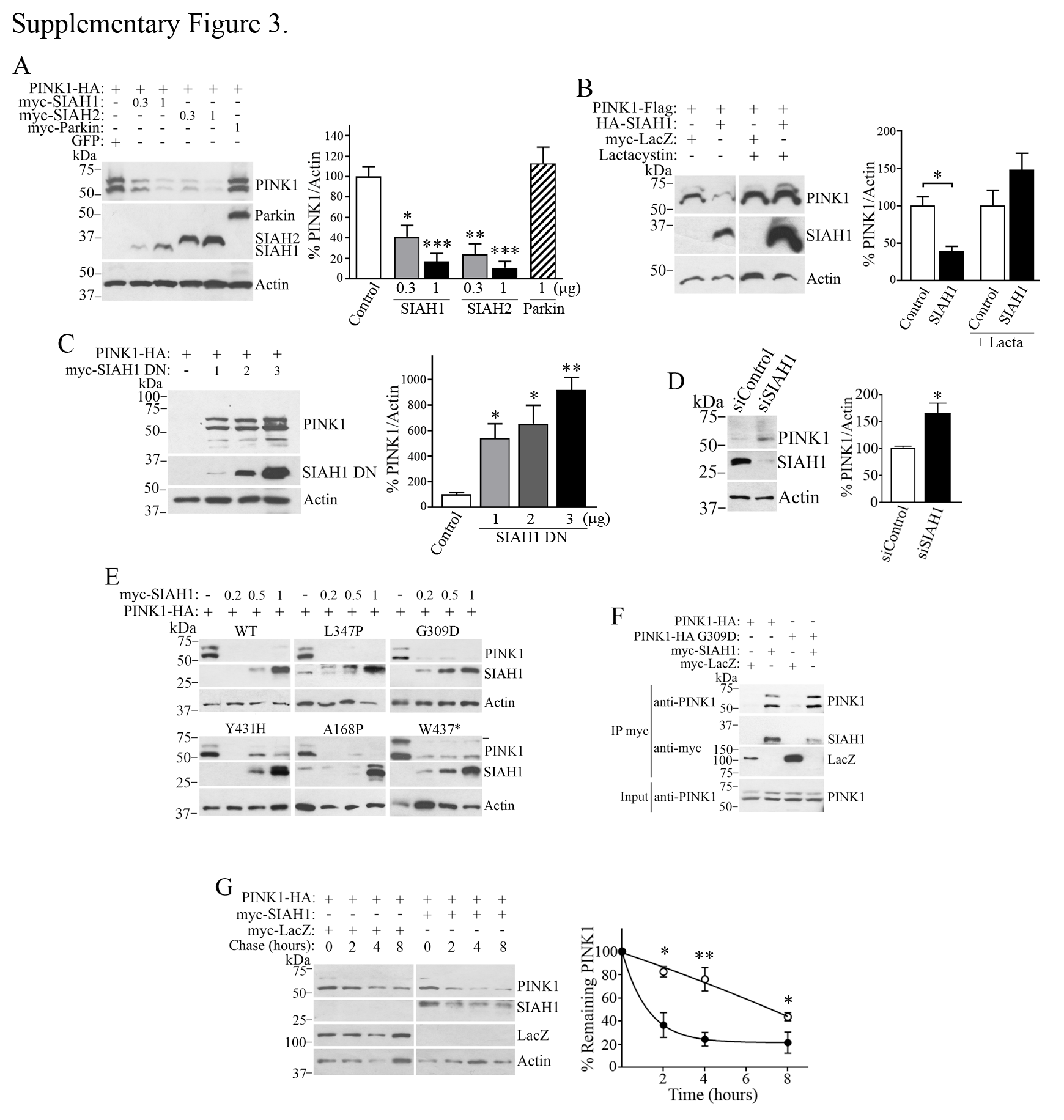

Supplement: Supplementary file 3 — Appendix S1 [file ACEL-21-e13731-s001.tif]

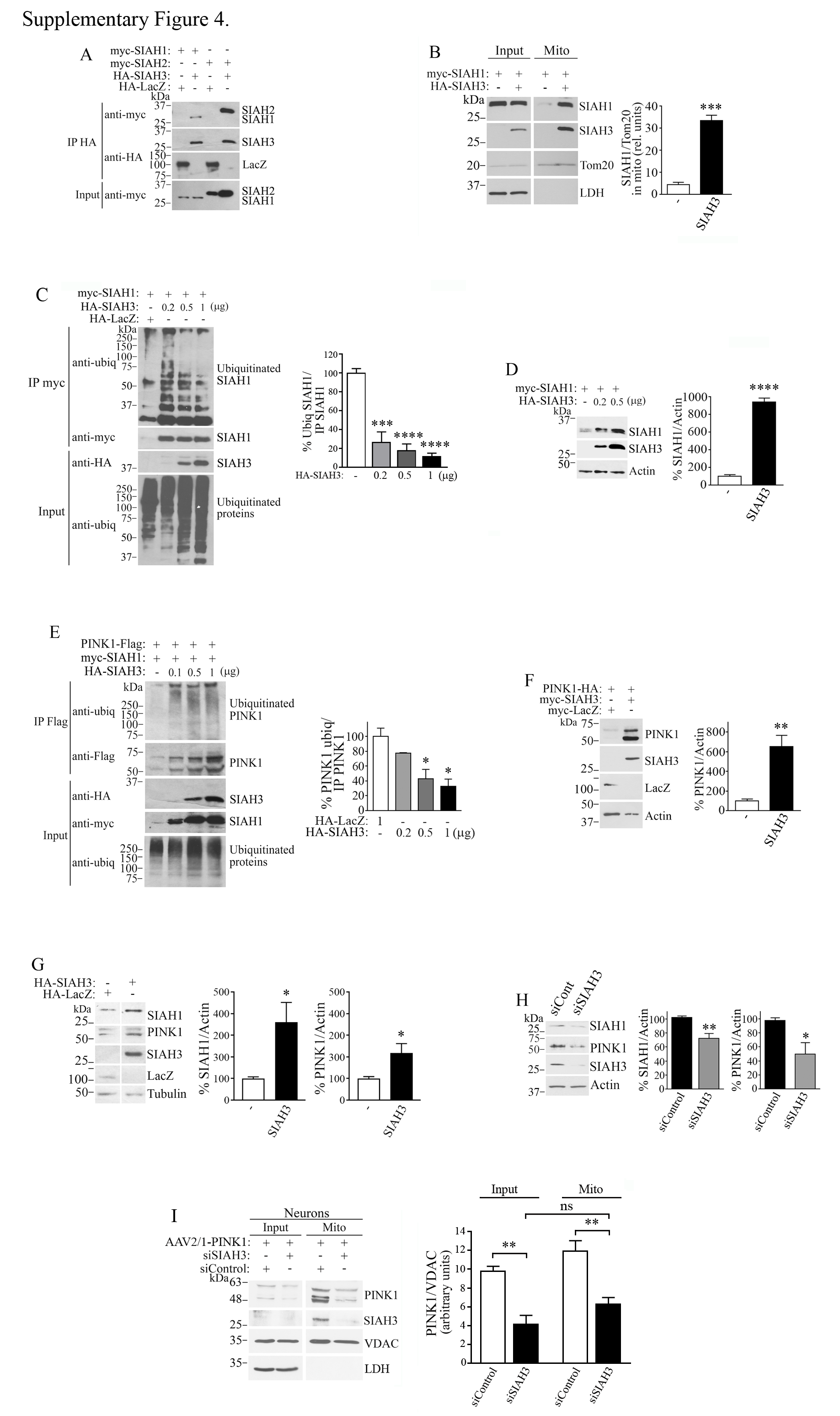

Supplement: Supplementary file 4 — Appendix S1 [file ACEL-21-e13731-s005.tif]
