# Supplementary material for: SIAH proteins regulate the degradation and intra‐mitochondrial aggregation of PINK1: Implications for mitochondrial pathology in Parkinson's disease
Source: Aging Cell. 2022 Oct 28;21(12):e13731. doi: 10.1111/acel.13731 (PMC9741505; doi:10.1111/acel.13731)
Supplement: Supplementary file 5 — Appendix S1 [file ACEL-21-e13731-s003.docx]

**Supplementary Figure 1. Characterization of α-SynPFF. A,** α-SynPFF (not sonicated and sonicated as described in Materials and Methods) were analyzed by transmission electron microscopy. Scale, 200 nm. **B,** Neurons were incubated with α-synuclein (monomeric) and sonicated α-SynPFF for 14 days. Neurons were assayed with JC-1 and analyzed by live microscopy. The presence of red JC-1 indicates polarization of mitochondrial neurons, whereas green JC-1 represents depolarization. Scale, 10 μm. Graph represents the ratio of red JC-1 relative to green, in the absence and presence of monomeric α-synuclein and α-SynPFF. Values represent the average ± SEM of 3 experiments. * Different from control at p= 0.0211 (Repeated measures one-way ANOVA with Bonferroni post-hoc test).

**Supplementary Figure 2. SIAH1 ubiquitinates PINK1 in HEK293 cells. A,** SIAH1 promotes the ubiquitination of PINK1. Transfected HEK293 cells were treated with 10 μM of the proteasome inhibitor lactacystin for 16h before harvesting. PINK1-HA was immunoprecipitated from transfected HEK293 cells with anti-HA, and levels of immunoprecipitate ubiquitination were determined using anti-ubiquitin. Graph represents the percent of ubiquitinated PINK1-HA relative to the levels of immunoprecipitated PINK1-HA. Values represent the average ± SEM of 3 experiments. * p= 0.046 (Student’s *t*-test). **B,** Ubiquitination of PINK1 by SIAH1 is processed by the proteasome only. Transfected HEK293 cells were treated with 10 μM lactacystin, 10 mM 3-MA (autophagy inhibitor), or 25 mM ammonium chloride (lysosomal inhibitor) for 16h before harvesting. PINK1-HA was immunoprecipitated from transfected HEK293 cells with anti-HA, and levels of immunoprecipitate ubiquitination were determined using anti-ubiquitin. Graph represents the percent of ubiquitinated PINK1-HA relative to the levels of immunoprecipitated PINK1-HA, in the presence of inhibitors to the different degradation pathways. Values represent the average ± SEM of 3 experiments. * p= 0.0335 (lactacystin), 0.0493 (3-MA) and 0.0276 (ammonium chloride) (Student’s *t*-test). **C,** SIAH1 knockdown decreases PINK1 ubiquitination. HEK293 cells were transfected in the presence of siRNA control or SIAH1 siRNA. PINK1-HA was immunoprecipitated with anti-HA, and levels of PINK1 ubiquitination were determined with anti-Flag. Graph represents the percent of ubiquitinated PINK1-HA relative to the levels of immunoprecipitated PINK1-HA. Values represent the average ± SEM of 3 experiments. * Different from control at p= 0.0318 (Student’s *t*-test).

**Supplementary Figure 3. Ubiquitination by SIAH1 promotes the proteasomal degradation of PINK1 in HEK293 cells. A,** SIAH1/2 reduce the steady-state levels of PINK1. Levels of PINK1 in transfected HEK293 cells were detected with anti-HA (upper panel). E3 ubiquitin-ligases were determined with anti-myc (middle panel). Graph represents the percent of PINK1 levels relative to actin, in the presence of SIAH1, SIAH3 and Parkin. Values represent the average ± SEM of 3 experiments. *, *** Different from control at p= 0.0102 and 0.0007 (SIAH1); **, *** p= 0.0015 and 0.0004 (SIAH2) (Repeated measures one-way ANOVA with Bonferroni post-hoc test). **B,** Transfected HEK293 cells were incubated in the absence and in the presence of 10 μM lactacystin for 16 hours. Levels of PINK1 were detected with anti-Flag (upper panel) and SIAH1 with anti-HA (middle panel). Graph represents the percent of PINK1 levels relative to actin, in the absence and in presence of SIAH1. Values represent the average ± SEM of 3 experiments. * Different from control at p= 0.0473 (Student’s *t*-test). **C,** HEK293 cells were transfected with PINK1-HA and increasing amounts of SIAH1 DN (catalytically inactive SIAH1; C55A, H59A, C72S). Levels of PINK1 were determined with anti-HA (upper panel) and SIAH1 DN with anti-myc (middle panel). Graph represents the percent of PINK1 levels relative to actin, in the presence of increasing amounts of SIAH1 DN. Values represent the average ± SEM of 3 experiments. *, ** Different from control at p= 0.0486 (1 μg), 0.0168 (2 μg) and 0.0016 (3 μg) (Repeated measures one-way ANOVA with Bonferroni post-hoc test). **D,** Endogenous PINK1 levels are increased upon SIAH1 knockdown. HEK293 cells were transfected with control siRNA or siRNA to SIAH1. PINK1 levels were determined with anti-PINK1 (upper panel) and knockdown of SIAH1 with anti-SIAH1 (middle panel). Graph depicts the percent of PINK1 steady-state levels relative to β-actin in the presence of the siRNAs. Values represent the average ± SEM of 3 independent experiments. * Different from control at p= 0.0263 (Student’s *t*-test). **E,** PINK1 disease mutants are equally degraded by SIAH1. Levels of PINK1 wild-type and mutants were detected with anti-HA (upper panel). E3 ubiquitin-ligases levels were determined with anti-myc (middle panel). **F,** Interaction of PINK1 G309D with SIAH3. SIAH1 was immunoprecipitated with anti-myc (second panel), and co-immunoprecipitation of PINK1 proteins was determined with anti-PINK1 antibody (first panel). **G,** Degradation rate of PINK1 increases by SIAH1. Transfected cells were incubated with cycloheximide 50 μg/ml to prevent protein synthesis and chased for indicated time points. Levels of remaining PINK1 were determined using anti-HA (first panels). Levels of SIAH1 and LacZ were detected with anti-myc (second and third panels, respectively). Graph represents the percent of remaining PINK1-HA, in the presence of SIAH1 or LacZ. Values represent the average ± SEM of 3 experiments. *,** Different from control at p=0.0343 (2 hrs), 0.0439 (8 hrs) and 0.0094, respectively (Student's *t*-test for each time point analyzed).

**Supplementary Figure 4. SIAH3 inhibits SIAH1 activity and the ability to degrade endogenous PINK1 in HEK293 cells. A,** SIAH1/2 interact with SIAH3 in cells. SIAH3 was immunoprecipitated with anti-HA (second panel), and co-immunoprecipitation of SIAH1 and 2 was determined with anti-myc (first panel). **B,** SIAH3 promotes the translocation of SIAH1 to the mitochondria. Mitochondrial fractions from transfected HEK293 were analyzed, and the presence of SIAH1 was determined with anti-myc (first panel). Levels of SIAH3 in mitochondrial was detected with anti-HA (second panel). The purity of the fractionation was determined by the levels of Tom20 and LDH in mitochondrial fractions (third and fourth panels). Graph represents the relative levels of SIAH1 in mitochondrial fractions corrected by the levels of Tom20, in the absence and the presence of SIAH3. Values represent the average ± SEM of 3 experiments. *** Different from control at p= 0.0005 (Student’s *t*-test). **C,** Decrease of SIAH1 auto-ubiquitination by SIAH3 in HEK293 cells. Levels of SIAH1 ubiquitination were determined with anti-ubiquitin (first panel). Immunoprecipitated SIAH1 was detected with anti-myc (second panel), while the increasing amount of SIAH3 was obtained with anti-HA (third panel). Graph represents the percent of ubiquitinated SIAH1 relative to immunoprecipitated SIAH1, in the presence of increasing SIAH3 amount. Values represent the average ± SEM of 3 experiments. ***, **** Different from control at p= 0.0002 and < 0.0001, respectively (Repeated measures one-way ANOVA with Bonferroni post-hoc test). **D,** Steady-state levels of SIAH1 in the presence of an increasing amount of SIAH3 was determined with anti-myc (first panel). Levels of expressed SIAH3 were determined with anti-HA (second panel). Graph represents the percent of SIAH1 relative to actin in the absence and presence of SIAH3 (0.5 μg). Values represent the average ± SEM of 3 experiments. **** Different from control at p< 0.0001 (Student’s *t*-test). **E,** Decrease of SIAH1-mediated PINK1 ubiquitination by SIAH3. Levels of PINK1 ubiquitination were determined with anti-ubiquitin (first panel). Immunoprecipitated PINK1 was detected with anti-Flag (third panel), while the increasing amount of SIAH3 was determined with anti-HA (fourth panel). Graph represents the percent of ubiquitinated PINK1 relative to immunoprecipitated PINK1, in the presence of increasing SIAH3 amount. Values represent the average ± SEM of 3 experiments. * Different from control at p= 0.0318 and 0.0157 (0.5 and 1 μg, respectively) (Repeated measures one-way ANOVA with Bonferroni post-hoc test). **F,** Steady-state levels of PINK1 in the absence and presence of SIAH3 were determined with anti-Flag (first panel). Levels of expressed SIAH3 were determined with anti-HA (second panel). Graph represents the percent of PINK1 relative to actin in the absence and presence of SIAH3. Values represent the average ± SEM of 3 experiments. ** Different from control at p= 0.0083 (Student’s *t*-test). **G,** Levels of endogenous SIAH1 and PINK1 in the presence of increasing SIAH3 were determined with anti-SIAH1 (first panel) and anti-PINK1 (second panel), respectively. Levels of expressed SIAH3 were determined with anti-HA (second panel). Graphs represent the percent of SIAH1 and PINK1 relative to actin in the presence of increasing SIAH3. Values represent the average ± SEM of 3 experiments. * Different from control at p= 0.0479 (for SIAH1 levels) and p= 0.0368 (for PINK1 levels) (Student’s *t*-test). **H,** Levels of endogenous SIAH1 and PINK1 in the presence of control siRNA and siRNA to SIAH3 were determined with anti-SIAH1 (first panel) and anti-PINK1 (second panel), respectively. Levels of endogenous SIAH3 were determined with anti-SIAH3 (third panel). Graphs represent the percent of SIAH1 and PINK1 relative to actin in the presence of siRNA control and to SIAH3. Values represent the average ± SEM of 3 experiments. For SIAH1 levels, ** different from control at p= 0.0059 (Student’s *t*-test). For PINK1 levels, * different from control at p= 0.0209 (Student’s *t*-test). **I,** Neurons were transduced with AVV2/1-PINK1 and transfected with siRNAs (control and SIAH3). Mitochondrial fractions were purified and the levels of PINK1 in input and mitochondrial fractions were compared using anti-PINK1. The purity of mitochondrial fractions were determined with anti-VDAC and anti-LDH. Graph represents the relative levels of PINK1 in input and mitochondrial fractions relative to VDAC, in the presence of siSIAH3 and siControl. Values represent the average ± SEM of 3 experiments. ** p= 0.0069 (input) and 0.0071 (mito); ns= 0.5949 (Repeated measures one-way ANOVA with Bonferroni post-hoc test).
