# Supplementary material for: SIAH proteins regulate the degradation and intra‐mitochondrial aggregation of PINK1: Implications for mitochondrial pathology in Parkinson's disease
Source: Aging Cell. 2022 Oct 28;21(12):e13731. doi: 10.1111/acel.13731 (PMC9741505; doi:10.1111/acel.13731)
Supplement: Supplementary file 6 — Appendix S1 [file ACEL-21-e13731-s002.docx]

**Materials and Methods (Supplementary Information)**

### **Cell culture and transfections**

HEK293 cells were grown in DMEM containing 10% fetal bovine serum in a 5% CO_2_ atmosphere. HEK293 cells were transiently transfected with N-terminal or C-terminal-tagged pRK5 plasmids utilizing Lipofectamine 2000 (Invitrogen). PINK1 K219M was used as kinase-dead ([Haskin et al., 2013](#_ENREF_15)). For experiments using small-interference RNAs, cells were transfected with Lipofectamine 2000 as previously described ([Avraham et al., 2007](#_ENREF_2)) using siSIAH1 (5'-CGCCCAUUCUUCAAUGUCA-3'), siSIAH3 (5'-GGCCGACUGCUUCACCUAU-3'), siPINK1 using siPINK1 (5'-GGCAAUUUUUACCCAGAAA-3'), or scrambled siRNA control (Ambion).

### **Antibodies and Western blot analysis**

Samples were homogenized as previously described ([Liani et al., 2004](#_ENREF_30)), run on 10% PAGE-SDS, and transferred to 0.2 μm nitrocellulose membranes. Membranes were blocked in 5% nonfat dried milk and 0.1% Tween 20, and then incubated with primary antibodies at room temperature for 1 hour. Blots were probed with the antibodies mouse anti-HA (901501, BioLegend); mouse anti-actin (SKU 08691001, MP Biomedicals); rabbit anti-myc (sc-789), rabbit anti-HA (sc-805), mouse anti-LDH (sc-133123), rabbit anti-Tom20 (sc-11415), mouse anti-HSP60 (sc-376261), mouse anti-GST (sc-138), mouse anti-ubiquitin (sc-8017), goat anti-PINK1(sc-32584), goat anti-SIAH1 (sc-5505)(Santa Cruz); mouse anti-myc (M4439), mouse anti-Flag (F1804), rabbit anti-Flag (F7425), rabbit anti-GAPDH (G9545); rabbit anti-α-synuclein (S3062) (Sigma); rabbit anti-VDAC (4866S, Cell Signaling); rabbit anti-ubiquitin (Z0458, Dako); rabbit anti-phopho-ubiquitin (Boston Biochem A-110); rabbit anti-PINK1 (BC100-494, Novus); goat anti-SIAH1 (ab2237), rabbit anti-SIAH3 (ab177739) (Abcam); mouse anti-α-synuclein (610787, BD Biosciences); mouse anti-phospho-S129-α-synuclein (pSyn#64, Wako). Additional rabbit polyclonal antibodies, including anti-PINK1, anti-SIAH1, anti-Parkin, and anti-synphilin-1 antibodies, were generated and purified as previously described (Engelender et al., 1999, Liani et al., 2004, Haskin et al., 2013, Szargel et al., 2016). HRP-secondary antibodies (Jackson Laboratory) were incubated in 5% nonfat dried milk and 0.1% Tween 20 for an additional hour and developed by luminol-based chemiluminescent substrate (SuperSignal West Dura, Thermo Fischer Scientific). Quantifications of enhanced chemiluminescence reactions were carried out according to ImageMaster analysis. For endogenous brain and HEK293 lysates, anti-SIAH3 antibody was incubated overnight at 4^o^C in 5% nonfat dried milk and 0.1% Tween 20.

**α-SynPFF generation and use in primary cultures**

Concentrated mouse α-SynPFF (StressMarq SPR-322) were sonicated 15 times for 2 seconds each time at 30% potency using the probe sonicator XL2020 (Heat Systems Inc). Five seconds were waited between every sonication. After sonication, α-SynPFF were diluted in fresh neurobasal medium (ThermoScientific) to a concentration of 4 μg/ml and added to neuronal cultures containing the same volume of medium, rendering the α-SynPFF final concentration to be 2 μg/ml. α-SynPFF was added to DIV4 of culture and incubated for 14 days. α-SynPFF was freshly sonicated every time it was added to neurons. After 14 days, neurons were processed for biochemistry or immunocytochemical assays. Monomeric α-synuclein 2 μg/ml was used as control for 14 days as well. Quality of α-synPFF was determined by transmission electron microscopy everytime a new batch α-synPFF was purchased (representative Figure S1A).

**AAV Production**

HEK293T cells were transfected with pAdDeltaF6 Helper (Addgene #112867), pAAV2/1  Rep/Cap  (Addgene #112862) and pAAV-hSyn-EGFP (Addgene #50465; synapsin promoter) in a combination of 2:2:1. SIAH3 and PINK1 open reading frames replaced EGFP to generate the different constructs used in the study (e.g., pAAV-hSyn-SIAH3 and pAAV-hSyn-PINK1). Transfections were carried out with PEI 1 mg/ml with a 1:1 ratio (1 μg DNA: 1 μl PEI). In general, ten 10 cm-plates were utilized for each recombinant virus. After 5 days of transfections, cells were processed as described (Groh et al., 2008). Briefly, cells were lysed in buffer containing 150mM NaCl, 50mM tris-HCL pH 8.5, and subjected to three freeze-and-thaw rounds. Lysates were treated with 100 U/ml Benzonaze (Sigma) for 1 hour at 37^0^C and further purified using iodixanol gradients (15-60%). Viruses were concentrated using Amicon filters (Merck) and stored at -80^0^C.

**Primary neuronal cultures**

E18 primary cortical cultures were prepared from Sprague-Dawley rats according to a protocol approved by the committee for animal experimentation at the Technion-Israel Institute of Technology (Protocol Number IL-021-02-20). Briefly, after decapitation, the cortices were retrieved and maintained in Hank's balanced salt solution. Then, the samples were digested by incubation for ten minutes with trypsin (Beit-Haemek Biological Industries) at 37ºC. This was followed by physical trituration with Pasteur pipettes, and the samples were briefly centrifuged for 10s at 1,000 x g to remove debris. The supernatant was filtered through a 0.7 µm filter (BD Biosciences), followed by centrifugation at 900 x g for 4 minutes. The cell pellet was resuspended in Neurobasal medium supplemented with B27 (Invitrogen) and 0.5mM L-glutamine (Beit-Haemek Biological Industries). Neurons were plated in 12 or 6 well plates covered with poly-D-Lysine (Sigma) at a density of 0.45 and 2 x10^6^ cells per well, respectively. Transfections were carried out at DIV4 using the calcium phosphate method, as previously described (Eyal et al., 2006), and analyzed for immunocytochemistry at DIV7. For siRNA transfections in neurons, 100 nM of siControl and siSIAH3 (Ambion) were transfected with Lipofectamine 2000 at DIV10 and processed for biochemistry at DIV14. For neuronal infections, recombinant AAV2/1 viruses (1x10^9^-1x10^10^ GC/ml) were added to the cultures at DIV10 and processed for biochemistry or immunocytochemistry at DIV17.

**Stereotaxic Injections**

Mice stereotaxic injections were done in C57Bl/6 mice according to a protocol approved by the committee for animal experimentation at the Technion-Israel Institute of Technology (Protocol Number IL-020-02-20). Immediately prior to the injections, mouse α-SynPFF (StressMarq SPR-322) were sonicated at power output of 30% for 15 times (2 seconds each time) with the probe sonicator XL2020 (Heat Systems Inc). Ten-week-old male were anesthetized with xylazine/ketamine and freshly sonicated α-SynPFF (2.5 μl/5 μg protein) was injected into the right striatum at the coordinates +0.5mm anterior, +2 mm lateral, and -3 mm relative to bregma (Paxinos and Franklin, 2004). Injections were done with pulled glass needle attached to a Hamilton syringe at a rate of 0.3μl per minute and after each injection, the needle was left in place for 5 min and then slowly withdrawn. Animals were frequently monitored following surgery and sacrificed after 30 days.

**Filter Trap Assays and Detergent extractions**

Purified mitochondria (5 μg) from transfected HEK293 cells, transduced cultured neurons and PD tissues were filtered on 0.2 μm pore size cellulose acetate membrane (Sartorius) as previously described ([Scherzinger et al., 1997](#_ENREF_44)). Membranes were then processed as described for Western blot analysis. Each filter trap experiment with transfected cells and transduced neurons was repeated three independent times. Filter trap assays of PD samples were repeated three independent times using every time newly prepared mitochondrial from the same set of human tissues.

For detergent extractions of total homogenates and isolated mitochondria, transfected HEK293 cells were incubated in buffer containing incremental SDS, urea and formic acid concentrations. Briefly, cells were lysed in buffer containing 50 mM Tris-HCl (pH 7.4), 140 mM NaCl, 1% Triton X-100, 2% SDS, 30 μM MG132, 20 mM NaF, 2 mM Na_3_VO_4_, 10 mM PPi, 20 mM β-glycerol phosphate, and protease inhibitor cocktail (MiniComplete, Roche). After 1 hour of incubation at room temperature, cell lysates were centrifuged for 10 min at 13,000xg. Pellet of cells were then resuspended in the same buffer with 1% Triton X-100, 2% SDS and 5M urea, and incubated for an additional hour at room temperature and centrifuged for 10 min at 13,000xg. Pellets were finally resuspended in buffer containing 1% Triton X-100, 2% SDS, 5M urea, and 70% formic acid, and incubated for an additional 30 min at 37^0^C. Samples with formic acid were neutralized with 1M Trizma base plus 10M NaOH. Neutralized samples were centrifuged for 10 min at 13,000xg, rendering insoluble pellets for 1% Triton X-100, 2% SDS, 5M urea, and 70% formic acid. All pellets were run on 10% PAGE-SDS and analyzed by Western blot.

For mice injected with α-SynPFF, striatum were homogenized in buffer containing 50 mM Tris-HCl (pH 7.4), 140 mM NaCl, 1% Triton X-100, 0.1% SDS, 30 μM MG132, 20 mM NaF, 2 mM Na_3_VO_4_, 10 mM PPi, 20 mM β-glycerol phosphate, and protease inhibitor cocktail (MiniComplete, Roche). After 1 hour of incubation at 4^0^C, striatum homogenates were centrifuged for 10 min at 13,000xg and separated into Triton/SDS-soluble and Triton/SDS-insoluble fractions.

**Binding experiments**

GST-SIAH3, GST-SIAH1, and PINK1-His constructs were expressed in *E. coli* BL21 bacteria and purified as described before ([Liani et al., 2004](#_ENREF_30)). PINK1-His proteins attached to His beads were incubated with purified GST-SIAH3 (2 mg/ml) in buffer containing 50 mM Tris-HCl (pH 7.4), 140 mM NaCl, 1% Triton X-100, 0.1% SDS, 1% DOC, 30 μM MG132, 20 mM NaF, 2 mM Na_3_VO_4_, 10 mM PPi, 20 mM β-glycerol phosphate, and protease inhibitor cocktail (MiniComplete, Roche). Binding was carried out at 4 ^0^C for 30 min and then washed in lysis buffer containing 500 mM NaCl and analyzed by Western blots.

### **Co-immunoprecipitation assays**

For the co-immunoprecipitations, transfected cells were lysed in buffer containing 50 mM Tris (pH 7.4), 140 mM NaCl, 1% Triton X-100, 0.1% SDS, 30 μM MG132, 20 mM NaF, 2 mM Na_3_VO_4_, 10 mM PPi, 20 mM β-Glycerol phosphate, and protease inhibitor cocktail (MiniComplete, Roche). Cell extracts were clarified by centrifugation and incubated for 4 hours with anti-HA (E6779), anti-myc (A7470), or anti-Flag (F2426) coupled to protein G beads (Sigma) ([Liani et al., 2004](#_ENREF_30)). Immunoprecipitates were washed with lysis buffer containing 500 mM NaCl and detected by Western blot.

For endogenous co-immunoprecipitation assays, rat brains were homogenized in lysis buffer as above. Brain homogenates were clarified by centrifugation at 13,000g for 5 min. 10 μg anti-PINK1 antibody (Santa Cruz) was coupled to protein G beads ([Liani et al., 2004](#_ENREF_30)) and incubated for 4 hours with brain homogenate (2 mg/ml). Immunoprecipitates were washed with lysis buffer and detected by Western blot using an anti-SIAH3 and anti-SIAH1 antibodies.

***In vivo* ubiquitination assays**

Transfected HEK293 cells and AAV2/1-PINK1-HA transduced neurons were processed as previously described ([Rott et al., 2008](#_ENREF_41)). Briefly, cells were directly lysed in buffer containing 50 mM Tris (pH 7.4), 140 mM NaCl and 1% SDS, and boiled at 100^o^C for 5 min. Next, the lysates were sonicated and ten times diluted with buffer containing 50 mM Tris (pH 7.4), 140 mM NaCl, 1% Triton X-100, 30 μM MG132, and protease inhibitor cocktail (Mini Complete, Roche). The samples were centrifuged at 13,000 x g for 5 min, and the supernatants were incubated with anti-tag antibodies coupled to beads (Sigma) for 4 hours at 4 ^o^C. For *in vivo* ubiquitination of SIAH1 in AAV2/1-SIAH1-transduced neurons, 10 μg SIAH1 antibody (Santa Cruz) was added to lysates and incubated 16h at 4^0^C followed by incubation with protein G (Pierce) for an additional 4 hours at 4^0^C. For both cells and neurons, beads were then extensively washed with buffer containing 50 mM Tris (pH 7.4), 500 mM NaCl, 1% Triton X-100, and 0.1 % SDS. Immunoprecipitates were loaded on an SDS-PAGE gel for Western blot analysis.

**Cycloheximide chase experiments**

Transfected cells were incubated with 50µM cycloheximide and harvested at the indicated time points. Cells were lysed in buffer containing 50mM Tris-HCl (pH 7.4), 140mM NaCl, 1% Triton X-100, 0.1% SDS, 30 μM MG132, and protease inhibitor cocktail (MiniComplete, Roche), and centrifuged for 5 minutes at 13,000xg. Lysates were analyzed by Western blot.

### **Immunocytochemistry**

Transfected and transduced neurons were fixed with 4% paraformaldehyde for 15 min and blocked in PBS containing 0.2% Triton X-100 and 5% normal goat serum. Neurons were labeled with primary antibodies as previously described ([Liani et al., 2004](#_ENREF_30)). Immunolabeling was detected using Cy^2^ and Cy^3^-labeled secondary antibodies (Jackson Laboratories). For SIAH3 and PINK1 distribution and mitochondrial shape and aggregation, samples were examined by AiryScan superresolution under a Zeiss LSM 880 confocal microscope. Optical sections were obtained under a 63x immersion objective at a definition of 1,024 x 1,024 pixels with the pinhole diameter adjusted to 1 μm. Sections were acquired under the same laser parameters and image magnification. Presence of SIAH3 and PINK1 aggregates in mitochondria was determined by an independent investigator blind to the conditions using ZEN Blue imaging software and according to Manders's co-localization coefficient ([Zinchuk et al., 2005](#_ENREF_64)). Nuclear condensation and fragmentation were determined by examining Hoechst 33342 staining. In each independent experiment, approximately 100 cells were examined in at least ten random fields for each condition, and the data are representative of at least three independent experiments.

**Live mitochondrial polarization analysis**

Mitochondrial membrane potential (ΔΨ_m_) was determined by JC-1 (5,5',6,6'-tetrachloro-1,1',3,3'-tetraethylbenzimidazolocarbocyanine iodide) (ThermoFischer Scientific) staining ([Ben-Shachar et al., 2015](#_ENREF_4)). HEK293 cells and neurons were incubated for 30 min at 37°C in 5% CO_2_ in the presence of 2.5 μg/ml JC-1. Live-cell images were acquired using Zeiss LSM 700 Laser Scanning Confocal System (excitation wavelength 488 nm, emission I at 530 ± 15 nm and emission II at 590 ± 20 nm) (Zeiss, Oberkochen, Germany) with 63/1.4× oil objective at a definition of 1,024 × 1,024 pixels and a pinhole diameter of 3 μm. Red fluorescence indicates JC-1 aggregates formed in cells with normal △ψm, whereas green fluorescence indicates JC-1 monomers in cells with low △ψm.

For cells, BFP (Blue Fluorescent Protein) alone and BFP with 10 μM CCCP (added 30 minutes before JC-1 incubation) were used as controls for polarized and depolarized mitochondria, respectively. At least 20 random fields were analyzed for each condition, and each experiment was repeated three times. All acquired images were analyzed by an independent investigator blind to the conditions. The number of completely green (depolarized) mitochondria was scored and divided by the total amount of BFP-positive cells.

For experiments with neurons, 2 μg/ml of sonicated α-SynPFF were added to cultures at DIV4 and processed for JC-1 after 14 days. No BFP was used in neurons. At least 20 random fields were captured for each condition, and each experiment was repeated three times. The ratio of red/green JC-1 was quantified in ZEN Blue imaging software by an independent investigator blind to the conditions.

**Human samples**

Post-mortem human brain tissues and CSF samples were obtained from Queen Square Brain Bank (QSBB, University College London). The samples were collected with informed consent of patient/next of kin and according to Ethics Committees’ guidelines. Final neuropathological diagnosis was ascertained by Neuropathologists affiliated to QSBB.

Brain samples were lysed in 10 volumes of buffer containing 50 mM Tris (pH 7.4), 140 mM NaCl, 1% Triton X-100, 0.1% SDS, 30 μM MG132, 20 mM NaF, 2 mM Na_3_VO_4_, 10 mM PPi, 20 mM β-Glycerol phosphate, and protease inhibitor cocktail (MiniComplete, Roche). Lysates were briefly sonicated and processed by Western blot. Samples (50 μg) were analyzed by Western blot three independent times. For each time, frozen brain samples were freshly homogenized and subjected to Western blot. The levels of SIAH3 were measured from a representative experiment.

CSF samples were stored at -80^0^C and were analyzed without any further additions. Additional samples with visible amounts of blood were excluded from the analysis to avoid confounding data from peripheral blood cells. PD and age-matched controls (3 of each) (20μl) were analyzed by Western blot three independent times. The levels of SIAH3 and α-synuclein were measured from a representative experiment.

**Demographics of human brains and CSF**

| **Substantia nigra** | M/F | Age (yrs) | PM delay (h) | Disease duration (years) |
| --- | --- | --- | --- | --- |
| iPD1 | M | 80 | 35.5 | 18.5 |
| iPD2 | F | 75 | 60 | 15 |
| iPD3 | F | 72 | 29 | 13.5 |
| iPD4 | M | 71 | 55.5 | 12 |
| iPD5 | M | 78 | 42.5 | 16.5 |
| Con1 | F | 90 | 32.5 | **-** |
| Con2 | F | 85 | 58 | **-** |
| Con3 | M | 78 | 48.5 | **-** |
| Con4 | M | 82 | 53 | **-** |
| Con5 | F | 75 | 39.5 | **-** |

| **Frontal Cortex** | M/F | Age (yrs) | PM delay (h) | Disease duration (years) |
| --- | --- | --- | --- | --- |
| iPD1 | M | 84 | 82.2 | 16 |
| iPD2 | M | 80 | 75.4 | 18 |
| iPD3 | M | 78 | 52 | 20 |
| iPD4 | F | 93 | 39.1 | 25 |
| Con1 | M | 69 | 40.35 | **-** |
| Con2 | F | 73 | 30.28 | **-** |
| Con3 | F | 84 | 71.5 | **-** |
| Con4 | M | 80 | 60 | **-** |

| **CSF** | M/F | Age (yrs) | PM delay (h) | Disease duration (years) |
| --- | --- | --- | --- | --- |
| PD1 | M | 74 | 20 | 25 |
| PD2 | M | 74 | 21 | 10 |
| PD3 | F | 78 | 16 | 26 |
| Con1 | M | 65 | 12 | - |
| Con2 | F | 84 | 11 | **-** |
| Con3 | F | 89 | 13 | **-** |

**Proteinase K assays**

Human substantia nigra were homogenized in ten volumes of buffer containing 50mM Tris (pH 7.4), 140 mM NaCl and 1% Triton X-100. Lysates were sonicated for 10 seconds and incubated with 1 μg/ml proteinase K for 30 minutes at 37^0^C, as described (Rott et al. 2017). Following digestion, lysates were analyzed by Western blot.

**Immunohistochemistry**

For immunohistochemistry, human midbrains were obtained from Queen Square Brain Bank (U.C.L., London). Paraffin-embedded sections (8 μm) of midbrain from three idiopathic PD and three control cases were deparaffinized in xylene followed by graded rehydration. Endogenous peroxidase activity was blocked with methanol/0.3% H2O2. Sections were pretreated by pressure cooking in citrate buffer at pH 6.0 for 10 min, and nonspecific protein binding was blocked in 10% normal goat serum in PBS for 30 min at room temperature. Double immunofluorescence was performed on all 5 iPD and 3 control midbrain sections, which were sequentially incubated with fluorescein and rhodhamine labeled secondary antibodies (Perkin Elmer) for visualizing SIAH3 and α-synuclein, respectively. Following adequate washes to remove background fluorescence, sections were mounted with Aquamount (Merck). Control sections where primary antibody was omitted displayed no significant background staining. Fluorescent signals of one midbrain section from every case were scanned using Leica fluorescent microscope (Leica CTR6000). Total α-synuclein-positive Lewy bodies were counted from each section, and the proportion of SIAH3 immunopositive Lewy bodies (LBs) were derived. For DAB stainings, adjacent sections of the same 5 iPD cases and 3 control cases were stained with anti-SIAH3 antibody. Primary antibodies were incubated at 4°C for 16 hours, immunostained by the avidin-biotin-peroxidase complex method antibody (Santa Cruz). Sections were counterstained with Mayer's hematoxylin.

**Electron microscopy**

HEK293 cells and neurons were transfected/transduced with PINK1 and SIAH3 constructs. After 36h, cells were washed with preheated serum-free medium, fixed for 1 hour at RT with 2% glutaraldehyde and 3% paraformaldehyde in 0.1 M sodium cacodylate buffer containing 5 mM CaCl_2_ and 3% sucrose. Cells were washed with sodium cacodylate buffer, scraped, and embedded in agarose. Following post-fixation and staining with 1% osmium tetraoxide, 0.5% potassium hexacyanoferrate, 0.5% potassium dichromate in 0.1 M cacodylate buffer, the samples were en-block stained with 1% uranyl acetate for 1 hour at RT Cells were then dehydrated in graded ethanol series, transferred to propylene oxide, and embedded in Epon 812. Ultrathin sections (75 nm) were cut with an ultramicrotome UC7 (Leica), transferred to copper grids (Pelco 1GNC200), and viewed using Talos L120C Transmission Electron Microscope at accelerating voltage of 120 keV. Transmission electron microscopy experiments were performed three independent times. For each experiment, two blocks of each condition were generated. Using sections from both blocks, at least 50 random fields were captured across different grid quadrants. Pictures were quantified for intra-mitochondrial aggregates in a blind manner by an independent examiner.

For immunoelectron microscopy, transfected cells were washed with preheated serum-free medium, fixed for 1 hour at RT with 0.1% glutaraldehyde and 4% paraformaldehyde in 0.1 M Sodium Cacodylate buffer for 1 hour at 4°c. Cells were washed with Sodium Cacodylate buffer, scraped, centrifuged at 500g for 3min, and small pieces of cell pellet were incubated in 2% aqueous uranyl acetate for 30 min. Then the cells were dehydrated in graded ethanol series and embedded in LR White resin. 75 nm ultrathin sections were cut with an ultramicrotome UC7 (Leica) and transferred to nickel grids (Pelco 1GN200). Following blocking with 1 % BSA, 0.5% FSG, 0. 1% glycine in PBS, the sections were incubated with primary and then with gold-labeled secondary antibodies (Jackson). After extensive washing, the sections were contrast stained with uranyl acetate. Finally, the grids were viewed using Talos L120C Transmission Electron Microscope at accelerating voltage of 120 keV. Immunoelectron microscopy experiments were performed three independent times. For each experiment, two blocks of each condition were generated. Using sections from both blocks, at least 50 random fields were captured across different grid quadrants. Pictures were then quantified for intra-mitochondrial aggregates in a blind manner by an independent examiner.

For the negative stainings to evaluate α-SynPFF, non-sonicated and sonicated samples were incubated on glow-­discharged Formvar/Carbon coated 200 mesh copper grids (Pelco 01800-F) for 10 min at RT. The grids were washed twice with distilled water and stained for 1 min with a 1% w/v uranyl acetate solution. The blotted grids were air-dried and imaged using Talos L120C Transmission Electron Microscope at accelerating voltage of 120 keV. For every experiment, at least 20 random fields were captured across different grid quadrants. Pictures were then quantified for α-SynPFF size using ImageJ analysis.

**Statistical Analysis**

Statistical analysis was performed by repeated measures one-way ANOVA with Bonferroni's multiple comparison test or two-tailed Student's t-test using GraphPad Prism software version 6.03 (GraphPad Inc.). The normality of human and mouse samples were determined by the Shapiro-Wilk test (GraphPad Prism 6.03) and considered normally distributed when p was greater than 0.05. Shapiro-Wilk test:

Fig.1A: p=0.0086 (SIAH3 Nigra Control 1A), p=0.8148 (SIAH3 Nigra PD 1A)

Fig. 1B: p=0.1883 (SIAH3 Cortex Control 1B), p=0.1255 (SIAH3 Cortex PD 1B)

Fig. 1C: p=0.1759 (SIAH3/α-Syn CSF Control 1C), p=0.4900 (SIAH3/α-Syn CSF PD 1C), p=0.0531 (SIAH3/Ponceau CSF Control 1C), p=0.1667 (SIAH3/α-Syn CSF PD 1C)

Fig. 2B: p=0.0512 (SIAH3 Nigra Control 2B), p=0.4857 (SIAH3 Nigra PD 2B), p=0.2605 (PINK1 Nigra Control 2B), p=0.2157 (PINK1 Nigra PD 2B), p=0.2010 (α-Syn Nigra Control 2B), p=0.8130 (α-Syn Nigra PD 2B)

Fig. 2C: p=0.1022 (SIAH3 contralateral striatum insoluble 2C), p=0.6990 (SIAH3 ipsilateral striatum insoluble 2C), p=0.6369 (PINK1 contralateral striatum insoluble 2C), p=0.2845 (PINK1 ipsilateral striatum insoluble 2C)

Fig. 2D: p=0.9868 (SIAH3 mito Control 2D), p=0.8871 (SIAH3 mito PD 2D), p=0.3927 (PINK1 mito Control 2D), p=0.8291 (PINK1 mito PD 2D).

All Western blots are representative of at least three independent experiments.

**Bibliography (Supplementary Information)**

Avraham, E., Rott, R., Liani, E., Szargel, R., & Engelender, S. (2007). Phosphorylation of Parkin by the Cyclin-dependent Kinase 5 at the Linker Region Modulates Its Ubiquitin-Ligase Activity and Aggregation. *J Biol Chem, 282*(17), 12842-12850. [doi.org/10.1074/jbc.M608243200](https://doi.org/10.1074/jbc.M608243200)

Ben-Shachar, D., Suss-Toby, E., & Robicsek, O. (2015). Analysis of mitochondrial network by imaging: proof of technique in schizophrenia. *Methods Mol Biol, 1265*, 425-439. doi:10.1007/978-1-4939-2288-8_32

Engelender, S., Kaminsky, Z., Guo, X., Sharp, A.H., Amaravi, R.K. Kleiderlein, J.J., …Ross, C.A. (1999). Synphilin-1 associates with alpha-synuclein and promotes the formation of cytosolic inclusions. *Nat Genet, 22*(1): 110-114. doi: 10.1038./8820

Eyal, A., Szargel, R., Avraham, E., Liani, E., Haskin, J., Rott, R., & Engelender, S. (2006). Synphilin-1A: an aggregation-prone isoform of synphilin-1 that causes neuronal death and is present in aggregates from alpha-synucleinopathy patients. *Proc Natl Acad Sci U S A, 103*(15), 5917-5922. doi: [10.1073/pnas.0509707103](https://doi.org/10.1073/pnas.0509707103)

Groh, A., de Kock, C.P.J., Wimmer, V.C., Sakmann, B., & Kuner, T. (2008). Driver or coincidence detector: Modal switch of a corticothalamic giant synapse controlled by spontaneous activity and short-term depression. *J Neurosci 28*(39), 9652-9663. doi: 10.1523/JNEUROSCI.1554-08.2008

Haskin, J., Szargel, R., Shani, V., Mekies, L. N., Rott, R., Lim, G. G., . . . Engelender, S. (2013). AF-6 is a positive modulator of the PINK1/parkin pathway and is deficient in Parkinson's disease. *Hum Mol Genet, 22*(10), 2083-2096. doi: [10.1093/hmg/ddt058](https://doi.org/10.1093/hmg/ddt058)

Liani, E., Eyal, A., Avraham, E., Shemer, R., Szargel, R., Berg, D., . . . Engelender, S. (2004). Ubiquitylation of synphilin-1 and alpha-synuclein by SIAH and its presence in cellular inclusions and Lewy bodies imply a role in Parkinson's disease. *Proc Natl Acad Sci U S A, 101*(15), 5500-5505. doi: [10.1073/pnas.0401081101](https://doi.org/10.1073/pnas.0401081101)

Rott, R., Szargel, R., Haskin, J., Shani, V., Shainskaya, A., Manov, I., . . . Engelender, S. (2008). Monoubiquitylation of alpha-synuclein by seven in absentia homolog (SIAH) promotes its aggregation in dopaminergic cells. *J Biol Chem, 283*(6), 3316-3328. doi: [10.1074/jbc.M704809200](https://doi.org/10.1074/jbc.m704809200)

Rott, R., Szargel, R., Haskin, J., Shani, V., Hamza, H., Savyon, M., . . . Engelender, S. (2017). SUMOylation and ubiquitination reciprocally regulate α-synuclein degradation and pathological aggregation. *Proc Natl Acad Sci U S A, 114*(50), 13176-13181. doi: 10.1073/pnas.1704351114

Scherzinger, E., Lurz, R., Turmaine, M., Mangiarini, L., Hollenbach, B., Hasenbank, R., . . . Wanker, E. E. (1997). Huntingtin-encoded polyglutamine expansions form amyloid-like protein aggregates in vitro and in vivo. *Cell, 90*(3), 549-558. doi:10.1016/s0092-8674(00)80514-0

Szargel, R., Shani, V., Abd Elghani, F., Meikes, L.N., Liani, E., Rott, R., & Engelender, S. (2016). The PINK1, synphilin-1 and SIAH-1 complex constitutes a novel mitophagy pathway. *Hum Mol Genet, 25*(16): 3476-3490. doi: 10.1093/hmg/ddw189

Zinchuk, V., Zinchuk, O., & Okada, T. (2005). Experimental LPS-induced cholestasis alters subcellular distribution and affects colocalization of Mrp2 and Bsep proteins: a quantitative colocalization study. *Microsc Res Tech, 67*(2), 65-70. doi:10.1002/jemt.20184
